# Significant reduction of activity retention in the kidneys via optimized linker sequences in radiohybrid-based minigastrin analogs

**DOI:** 10.1186/s13550-024-01087-5

**Published:** 2024-03-02

**Authors:** Nadine Holzleitner, Sebastian Fischer, Isabel Maniyankerikalam, Roswitha Beck, Constantin Lapa, Hans-Jürgen Wester, Thomas Günther

**Affiliations:** 1 TUM School of Natural Sciences, Department of Chemistry, Chair of Pharmaceutical Radiochemistry, Technical University of Munich, Garching, Germany; 2 Nuclear Medicine, Faculty of Medicine, University of Augsburg, Augsburg, Germany; 3 Bavarian Cancer Research Center (BZKF), Bavaria, Germany

**Keywords:** Cholecystokinin-2 receptor (CCK-2R), Cholecystokinin-B receptor (CCK-BR), Medullary thyroid carcinoma (MTC), Minigastrin, Radiohybrid, rhCCK

## Abstract

**Background:**

We recently introduced radiohybrid (rh)-based minigastrin analogs e.g., DOTA-rhCCK-18 (DOTA-D-Dap(p-SiFA)-(D-γ-Glu)_8_-Ala-Tyr-Gly-Trp-Nle-Asp-Phe-NH_2_), that revealed substantially increased activity retention in the tumor. However, one major drawback of these first generation rh-based cholecystokinin-2 receptor (CCK-2R) ligands is their elevated activity levels in the kidneys, especially at later time points (24 h p.i.). Therefore, this study aimed to reduce kidney retention with regard to a therapeutic use via substitution of negatively charged D-glutamic acid moieties by hydrophilic uncharged polyethylene glycol (PEG) linkers of various length ((PEG)_4_ to (PEG)_11_). Furthermore, the influence of differently charged silicon-based fluoride acceptor (SiFA)-moieties (p-SiFA: neutral, SiFA-ipa: negatively charged, and SiFAlin: positively charged) on in vitro properties of minigastrin analogs was evaluated. Out of all compounds evaluated in vitro, the two most promising minigastrin analogs were further investigated in vivo.

**Results:**

CCK-2R affinity of most compounds evaluated was found to be in a range of 8–20 nM (by means of apparent *IC*_50_), while ligands containing a SiFA-ipa moiety displayed elevated *IC*_50_ values. Lipophilicity was noticeably lower for compounds containing a D-γ-glutamate (D-γ-Glu) moiety next to the D-Dap(SiFA) unit as compared to their counterparts lacking the additional negative charge. Within this study, combining the most favorable CCK-2R affinity and lipophilicity, [^177/nat^Lu]Lu-DOTA-rhCCK-70 (DOTA-D-Dap(p-SiFA)-D-γ-Glu-(PEG)_7_-D-γ-Glu-(PEG)_3_-Trp-(*N-*Me)Nle-Asp-1-Nal-NH_2_; *IC*_50_: 12.6 ± 2.0 nM; log*D*_7.4_: − 1.67 ± 0.08) and [^177/nat^Lu]Lu-DOTA-rhCCK-91 (DOTA-D-Dap(SiFAlin)-D-γ-Glu-(PEG)_4_-D-γ-Glu-(PEG)_3_-Trp-(*N*-Me)Nle-Asp-1-Nal-NH_2_; *IC*_50_: 8.6 ± 0.7 nM; log*D*_7.4_ =  − 1.66 ± 0.07) were further evaluated in vivo. Biodistribution data of both compounds revealed significantly reduced (p < 0.0001) activity accumulation in the kidneys compared to [^177^Lu]Lu-DOTA-rhCCK-18 at 24 h p.i., leading to enhanced tumor-to-kidney ratios despite lower tumor uptake. However, overall tumor-to-background ratios of the novel compounds were lower than those of [^177^Lu]Lu-DOTA-rhCCK-18.

**Conclusion:**

We could show that the reduction of negative charges within the linker section of radiohybrid-based minigastrin analogs led to decreased activity levels in the kidneys at 24 h p.i., while maintaining a good tumor uptake. Thus, favorable tumor-to-kidney ratios were accomplished in vivo. However, further optimization has to be done in order to improve tumor retention and general biodistribution profile.

**Graphical abstract:**

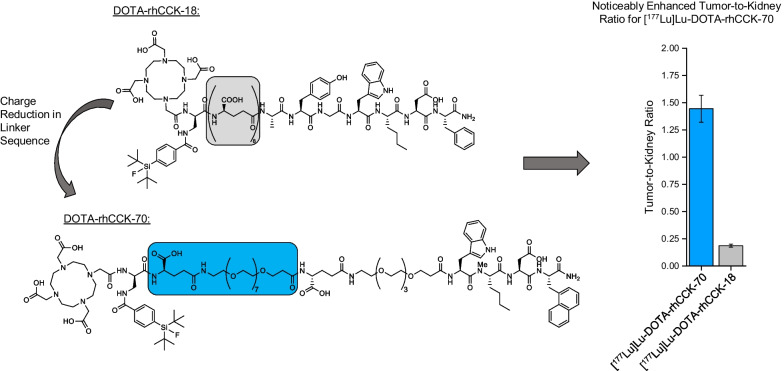

**Supplementary Information:**

The online version contains supplementary material available at 10.1186/s13550-024-01087-5.

## Introduction

Even though medullary thyroid carcinoma (MTC) is a rather rare form of thyroid disease [1], limited 10-year survival rates of less than 40% for patients suffering from locally advanced or progressive disease lead to a growing clinical interest in novel therapeutic approaches [2, 3]. Thus, over the past three decades research on peptide-based radiopharmaceuticals targeting the cholecystokinin-2 receptor (CCK-2R), which is overexpressed in over 90% of all MTC patients [4], has been progressing.

Only in 2019, the minigastrin analog DOTA-PP-F11N (DOTA-(D-Glu)_6_-Ala-Tyr-Gly-Trp-Nle-Asp-Phe-NH_2_) was evaluated ^177^Lu-labeled in clinical trials as one of the first lead structures for radioligand therapy of MTC [5, 6]. In addition, final results of a GRAN-T-MTC phase I clinical trial of the structurally similar compound [^111^In]In-CP04 (DOTA-(D-Glu)_6_-Ala-Tyr-Gly-Trp-Met-Asp-Phe-NH_2_) were recently published. Compared to conventional imaging strategies of MTC, such as 2-deoxy-2-[^18^F]fluoro-D-glucose (= [^18^F]FDG) as well as 6-[^18^F]-L-fluoro-L-3, 4-dihydroxyphenylalanine (= [^18^F]F-DOPA) positron emission tomography (PET)/computed tomography (CT), equivalent, complementary as well as superior performance of [^111^In]In-CP04 single-photon emission computed tomography (SPECT)/CT was reported in 16 patients, underlining the high potential of theranostic minigastrin analogs for patient management [7]. However, moderate metabolic stability and accelerated clearance kinetics of both DOTA-PP-F11N and CP04 limit their therapeutic efficacy [7–10]. One approach to circumvent stability issues in situ is the co-administration of [^177^Lu]Lu-DOTA-PP-F11N in combination with neutral endopeptidase (NEP)-1 inhibitors such as sacubitril, which has been evaluated in clinical trials with pending results (NCT03647657), while another theranostic clinical study using [^177^Lu]Lu-DOTA-PP-F11N (NCT02088645) is currently recruiting [11, 12].

Another approach is stabilization by chemical design, which was done for DOTA-PP-F11N and led to DOTA-MGS5 (DOTA-D-Glu-Ala-Tyr-Gly-Trp-(*N*-Me)Nle-Asp-1-Nal-NH_2_), a minigastrin analog comprising 1-Nal instead of Phe and (*N*-Me)Nle instead of Nle, as well as only one D-Glu moiety in the linker section [13]. Due to its high CCK-2R affinity accompanied by a favorable biodistribution profile in mice [13], first clinical results of [^68^ Ga]Ga-DOTA-MGS5 looked promising in MTC patients [14, 15]. Very recently, we further modified the DOTA-MGS5 sequence in order to address the Gly-Trp cleavage site, which resulted in DOTA-CCK-66 (DOTA-D-γ-Glu-(PEG)_3_-Trp-(*N-*Me)Nle-Asp-1-Nal-NH_2_), a simplified minigastrin analog displaying higher metabolic stability and thus, improved activity clearance and tumor-to-background ratios in animals, and which has already been successfully translated into the clinic [16, 17].

However, all compounds mentioned above are limited to radiometallation and do not allow for ^18^F-labeling, lacking the benefits of ^18^F-based PET [18]. In order to design an ^18^F-labeled minigastrin analog, we transferred the radiohybrid (rh) concept, which was successfully applied for prostate-specific membrane antigen-targeted compounds [19], to CCK-2R ligands in a previous study. This resulted in DOTA-rhCCK-18 (DOTA-D-Dap(p-SiFA)-(D-γ-Glu)_8_-Ala-Tyr-Gly-Trp-Nle-Asp-Phe-NH_2_), a rh-based minigastrin analog that enables both ^177^Lu- and ^18^F-labeling [20]. Both µSPECT/CT and µPET/CT imaging studies of the chemical identical compounds [^19^F]F-[^177^Lu]Lu-DOTA-rhCCK-18 as well as [^18^F]F-[^nat^Lu]Lu-DOTA-rhCCK-18, respectively, confirmed a similar biodistribution pattern in AR42J tumor-bearing mice at 1 h p.i. Compared to earlier generations of minigastrin analogs e.g., [^177^Lu]Lu-DOTA-MGS5 and [^177^Lu]Lu-DOTA-PP-F11N, [^177^Lu]Lu-DOTA-rhCCK-18 displayed 2- to 13-fold increased activity levels in the CCK-2R positive AR42J tumor at 24 h p.i. However, this was accompanied by unfavorably increased activity uptake in the kidneys, most likely due to the charge distribution in proximity to the silicon-based fluoride acceptor (SiFA) moiety [20]. Worth mentioning, we suspect a synergistic effect of the SiFA unit and the several negative charges in its proximity to be responsible for the substantial kidney retention observed for our previously developed rh-based CCK-2R-targeted (rhCCK) compounds such as [^177^Lu]Lu-DOTA-rhCCK-18 [8, 20]. We believe so, as [^177^Lu]Lu-DOTA-PP-F11N, which comprises a similar peptide sequence and a similar number of negative charges, did not reveal elevated activity levels in the kidneys at 24 h p.i. [8].

**Fig. 1:**
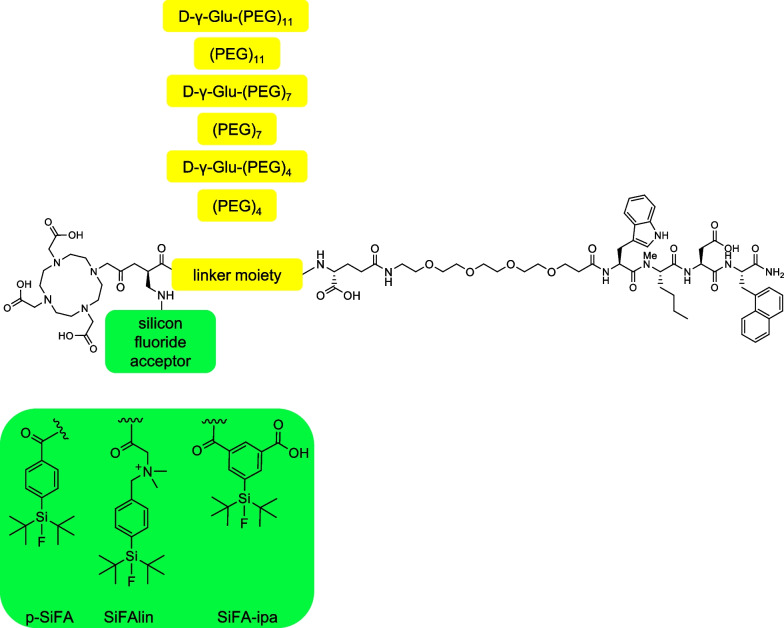
General composition of minigastrin analogs evaluated in this study. Yellow: linker sequences; Green: SiFA moieties

Hence, in this study we aimed to reduce kidney retention of rhCCK derivatives, while maintaining high activity levels in the tumor, with a strong focus on a potential therapeutic application. The presence of a poly-L-glutamate chain of minigastrin analogs has already been described as one of the main reasons for high kidney accumulation, which has successfully been addressed by various groups via the substitution of said chain by uncharged amino acids or PEG linkers [21, 22]. Therefore, we focused on the substitution of the poly-D-γ-glutamate linker section of our previously developed rhCCK derivatives by hydrophilic, uncharged PEG linkers of various length (4 to 11). Comparative evaluation of PEG linkers in combination with or without a D-γ-Glu moiety in proximity to the D-Dap(p-SiFA) building block (Fig. 1) was performed to assess the influence of an additional D-γ-Glu on the lipophilicity of the peptides, as compounds from previous works benefited from the introduction of the D-γ-Glu moiety with regard to metabolic stability and lipophilicity [16]. Worth mentioning, due to the high lipophilic character and the bulkiness of the SiFA building blocks, it is a challenge to maintain both high CCK-2R affinity and low lipophilicity for the whole compound. On the one hand, we observed that the distance between the SiFA moiety and the binding unit should be large to achieve high CCK-2R affinity [8]. On the other hand, previous works demonstrated that a lot of hydrophilic units are required to compensate the high lipophilicity of the p-SiFA group.

In order to reduce lipophilicity of the SiFA building block itself, thus demanding less hydrophilic moieties to compensate, we additionally evaluated the influence of negatively and positively charged SiFA moieties. We introduced either the SiFAlin moiety (positively charged), which was already used in somatostatin-based compounds [23], or 5-(di-*tert*-butylfluorosilyl)isophthalic acid (SiFA-ipa, negatively charged), which was recently developed in our group (Fischer et al., unpublished data). In order to ensure high general CCK-2R affinity and metabolic stability, we used the stabilized peptide sequence of DOTA-CCK-66 (*H*–D-γ-Glu-(PEG)_3_-Trp-(*N-*Me)Nle-Asp-1-Nal-NH_2_) previously developed in our group for all compounds evaluated [16]. In this study we evaluated the effect of the different modifications on in vitro properties of our compounds first, and later on in vivo properties at 24 h p.i for the two most promising ligands, especially with regard to kidney retention.

## Materials and methods

Evaluation of peptide identity and integrity is provided in the Additional file 1 (Fig. S1–S12). An expression^L^ CMS mass spectrometer (Advion Ltd., Harlow, UK) was used for characterization of the substances.

### Chemical synthesis and labeling procedures

Synthesis of the compounds was conducted as previously published [8, 20]. In brief, peptides were synthesized via standard fluorenylmethoxycarbonyl (Fmoc)-based solid phase peptide synthesis (SPPS) protocols using a *H-*Rink Amide ChemMatrix® resin (35–100 mesh particle size, 0.4–0.6 mmol/g loading, Merck KGaA, Darmstadt, Germany).

(4-(Bromomethyl)phenyl)di-*tert*-butylfluorosilane (SiFA-Br), which was used for generating the SiFAlin building block, was synthesized according to a published protocol [23]. Synthesis of 4-(di-tert-butylfluorosilyl)benzoic acid (p-SiFA) was completed according to an established protocol [24]. Chemical synthesis of the SiFA-ipa moiety is described in the Additional file 1 (Scheme S1). Coupling of p-SiFA was conducted in analogy to amino acid couplings to the side chain of a D-2,3-diaminopropionic acid (D-Dap). Conjugation of the SiFA-ipa moiety to the D-Dap side chain was accomplished similarly, yet using a threefold excess of SiFA-ipa to prevent dimerization. A threefold excess of SiFA-Br dissolved in CH_2_Cl_2_ was used to conjugate it to the *N-*terminus of *N*,*N*-dimethylglycine under basic conditions, resulting in a SiFAlin moiety.

^nat/177^Lu-Labeling of the peptide precursors was carried out according to protocols described in detail in the Additional file 1. In brief, ^nat^Lu-labeling was performed by adding an excess of [^nat^Lu]LuCl_3_ to the peptide precursor dissolved in H_2_O at 90 °C for 15 min. A final concentration of 0.1 nM of the ^nat^Lu-labeled peptide was achieved by the addition of H_2_O. ^177^Lu-labeling was conducted at 80 °C within 10 min (1.0 M NaOAc buffer, pH = 5.5).

### In vitro* experiments*

CCK-2R affinity (by means of apparent half-maximal inhibitory concentrations; *IC*_50_; using AR42J cells, 3 h incubation at 37 °C, 10^–11^ to 10^–5^ M ^nat^Lu-labeled peptide (in triplicate), 0.3 pmol [^177^Lu]Lu-DOTA-PP-F11N as radiolabeled reference, *n* = 3) as well as lipophilicity (expressed as n-octanol/phosphate buffered saline (PBS) (1/1) distribution coefficient; log*D*_7.4_, 1.0 MBq ^177^Lu-labeled peptide, *n* ≥ 5) were determined as previously published [8, 20].

For affinity determination, AR42J cells (2.0 × 10^5^ cells/well) were seeded into 24-well plates 24 ± 2 h prior to the experiment adding 1 mL of nutrient medium (RPMI 1640, 5 mM L-Gln, 5 mL non-essential amino acids (100 ×), 10% FCS) and incubating the well plates at 37 °C in a humidified atmosphere (5% CO_2_). On the next day, medium was removed, cells were washed with PBS (300 µL) and fresh nutrient medium supplemented with 5% BSA (200 µL) was added. The peptide of interest (25 µL in nutrient medium) in increasing concentrations (10^−10^ to 10^−4^ M) in triplicate as well as [^177^Lu]Lu-DOTA-PP-F11N (25 μL, 0.3 pmol) were added to the cells and the assay was incubated for 3 h at 37 °C. Subsequently, the supernatant was collected, cells were washed with PBS (300 µL) and both fractions were unified. Cell lysis was conducted by addition of NaOH (300 µL, 1 N) and incubation for at least 20 min at room temperature. The supernatant was collected, the respective wells were washed with NaOH (300 µL, 1 N) and both fractions were unified. Radioactivity of all fractions collected was quantified using a γ-counter (PerkinElmer Inc., Waltham, United States). Apparent half-maximal inhibitory concentration (*IC*_50_) was calculated via the GraphPad PRISM software (GraphPad Software Inc., La Jolla, United States).

For determination of lipophilicity, the respective ^177^Lu-Labeled peptide precursor (~ 1 MBq, 10 µL in 0.04 M HCl) was added to a solution of *n-*octanol/PBS (1/1, *v*/*v*, 1 mL) and vigourously mixed for 3 min at room temperature (*n* ≥ 5). Afterwards both phases were seperated using a Biofuge 15 centrifuge (Heraus Sepatech GmbH, Osterode, Germany) at 9,000 rpm for 5 min. 200 µL aliquots of both layers were collected separately, measured in a γ-counter (Perkin Elmer, Waltham, MA, USA) and the log*D*_7.4_ value was obtained.

Human serum albumin (HSA) binding was determined via high performance affinity chromatography (HPAC), according to previously published protocols [16, 25, 26]. Human serum albumin interaction was calculated according to the retention time (*n* = 1) of our compounds on a Chiralpak HSA column in dependence of nine reference compounds with known HSA interaction.

A detailed description of all in vitro experiments can be found in the Additional file 1.

### In vivo* experiments*

Animal experiments were carried out according to the general animal welfare regulations in Germany (German animal protection act, in the edition of the announcement, dated 18 May 2006, as amended by Article 280 of 19 June 2020, approval no. ROB-55.2–1-2532.Vet_02-18–109 by the General Administration of Upper Bavaria) and the institutional guidelines for the care and use of animals. Therefore, CB17-SCID mice of both genders and aged 2–4 months (Charles River Laboratories International Inc., Sulzfeld, Germany) were used. After arrival at the in-house facilities, mice were allowed to acclimate for a minimum of one week before inoculation of AR42J cells. AR42J cells (6.0 × 10^6^ cells per 200 μL) were suspended in a mixture (*v*/*v* = 1/1) of nutrient medium and Cultrex® Basement Membrane Matrix Type 3 (Trevigen Inc., Gaithersburg, MD, USA) and inoculated subcutaneously onto the right shoulder of CB17-SCID mice according to a previously reported protocol [8]. Animals were excluded from the study when reaching one of the following endpoints: a weight loss higher than 20%, a tumor size above 1500 mm^3^, an ulceration of the tumor, respiratory distress or change of behavior [8]. None of these criteria applied to any animal from the experiment. Neither randomization nor blinding was applied in the allocation of the experiments. Health status of the animals is specific pathogen free according to Federation of European Laboratory Animal Science Associations recommendation.

Biodistribution studies (*n* = 4) and µSPECT/CT imaging (*n* = 1) at 24 h p.i. were carried out as previously published [8]. For all in vivo experiments, approximately 2–3 MBq (100 pmol) of the respective ^177^Lu-labeled peptide were administered.

Acquired data were statistically analyzed by performing a Student’s *t*-test via Excel (Microsoft Corporation, Redmond, WA, USA) and OriginPro software (version 9.7) from OriginLab Corporation (Northampton, MA, USA). Acquired *p* values of less than 0.05 were considered statistically significant.

## Results

### Synthesis and radiolabeling

Fmoc-based SPPS with concomitant purification via reversed phase high performance liquid chromatography (RP-HPLC) yielded 5–20% peptide precursor (chemical purity > 95%, determined by RP-HPLC at λ = 220 nm). Quantitative ^nat^Lu-labeling was performed at 90 °C for 15 min using a 2.5-fold excess of [^nat^Lu]LuCl_3._ No further purification step prior usage was required, as the remaining free Lu^3+^ was shown to have no impact on *IC*_50_ determinations [27]. ^177^Lu-Labeling of all compounds resulted in quantitative radiochemical yields (RCY), radiochemical purities (RCP) higher than 95% as well as molar activities (A_m_) of 30 ± 10 GBq/µmol. Confirmation of peptide integrity and quality controls are provided in the Additional file 1 (Fig. S1–12). The names as well as the peptide structures of all compounds evaluated is shown in Table 1.

**Table 1:**
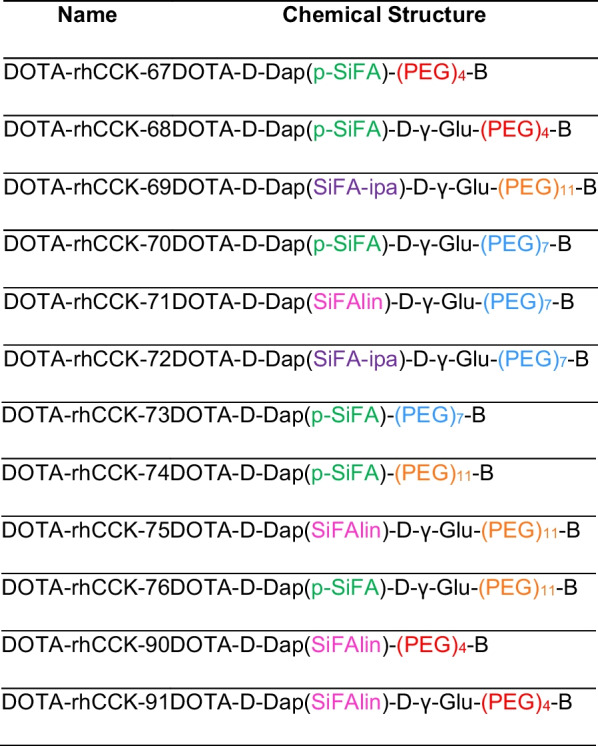
Names and peptide structures of all novel radiohybrid-based minigastrin analogs evaluated within this study (colour figure online)

All compounds comprise the binding unit B = D-γ-Glu-(PEG)_3_-Trp-(*N*-Me)Nle-Asp-1-Nal-NH_2_ as well as a DOTA chelating moiety. p-SiFA is depicted in green, SiFAlin in pink and SiFA-ipa in purple, whereas (PEG)_4_ is colored in red, (PEG)_7_ in blue and (PEG)_11_ in orange.

### In vitro* characterization*

**Fig. 2:**
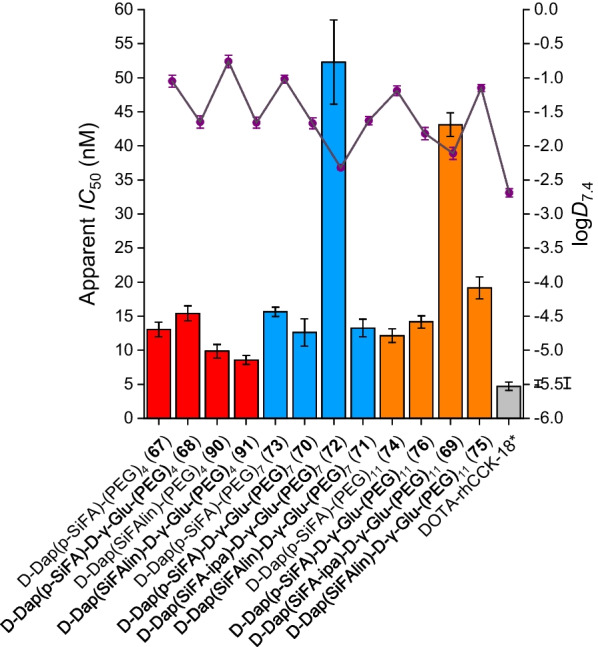
Affinity (apparent *IC*_50_) data (depicted in bars) and lipophilicity (log*D*_7.4_) data (depicted in purple dots) of the PEG_4_ containing compounds, [^nat/177^Lu]Lu-DOTA-rhCCK-67 -68, -90 and -91 (red), the PEG_7_ containing compounds, [^nat/177^Lu]Lu-DOTA-rhCCK-70 to -73 (blue), as well as the PEG_11_ containing compounds, [^nat/177^Lu]Lu-DOTA-rhCCK-74 to -76 and -69 (orange), compared to the reference [^nat/177^Lu]Lu-DOTA-rhCCK-18 (grey, [20]). All novel compounds comprise a [^177/nat^Lu]Lu-DOTA complex as well as a D-γ-Glu-(PEG)_3_-Trp-(*N*-Me)Nle-Asp-1-Nal-NH_2_ binding unit linked together by a spacer sequence X (defined on the X-axis). * data taken from Günther et al. [20]. These data have been determined in our lab under identical conditions

Affinity and lipophilicity data of all compounds evaluated are summarized in Fig. 2 and Additional file 1: Table S1.

Apparent *IC*_50_ values of most rh-based minigastrin analogs evaluated ([^nat^Lu]Lu-DOTA-rhCCK-67, − 68, − 70, − 71, − 73 to − 76 and − 90) were found to be in a range between 10 to 20 nM. For compounds containing a SiFA-ipa moiety ([^nat^Lu]Lu-DOTA-rhCCK-69 and -72), noticeably increased apparent *IC*_50_ values were observed. [^nat^Lu]Lu-DOTA-rhCCK-91, comprising a PEG_4_ linker in combination with a SiFAlin building block, displayed the highest CCK-2R affinity within this study (apparent *IC*_50_ = 8.56 ± 0.66 nM). However, compared to the reference compound, [^nat^Lu]Lu-DOTA-rhCCK-18 (apparent *IC*_50_ = 4.71 ± 0.62 nM, [20]), CCK-2R affinity of **91** was significantly decreased (*p* < 0.0014).

In general, all compounds lacking a D-γ-Glu moiety in proximity to the SiFA building block revealed a significantly higher lipophilicity than their counterparts comprising a D-γ-Glu moiety in said position (log*D*_7.4_ = –1.2 to − 0.8 vs. − 1.9 to –1.6; *p* < 0.0001), with [^177^Lu]Lu-DOTA-D-Dap(SiFAlin)-D-γ-Glu-(PEG)_11_-D-γ-Glu-(PEG)_3_-Trp-(*N*-Me)Nle-Asp-1-Nal-NH_2_ being the only exception. In addition, compounds containing a SiFA-ipa building block displayed the lowest lipophilicity among all compounds (log*D*_7.4_: − 2.4 to − 2.1), which was found to be slightly higher than that of [^177^Lu]Lu-DOTA-rhCCK-18 (log*D*_7.4_ = –2.71 ± 0.04, [20]).

HSA binding was found to be in a range between 85 and 95% for all compounds evaluated (Table S1). Except for [^nat^Lu]Lu-DOTA-rhCCK-75, an extended PEG linker length led to slightly reduced HSA binding. No trends regarding the influence of different silicon-based fluoride acceptors on HSA interaction were noticed. In comparison, the reference compound, [^nat^Lu]Lu-DOTA-rhCCK-18 (87.1%), displayed similar HSA binding to the novel rhCCK derivatives.

### In vivo* characterization*

**Fig. 3:**
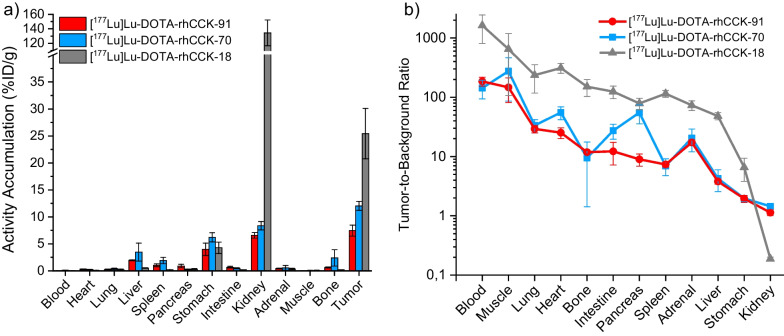
**a** Biodistribution data and **b** tumor-to-background ratios of [^177^Lu]Lu-DOTA-rhCCK-91 (red) and [^177^Lu]Lu-DOTA-rhCCK-70 (blue) in selected organs (depicted in percentage injected dose per gram; %ID/g) at 24 h p.i. in comparison to [^177^Lu]Lu-DOTA-rhCCK-18 (grey, [20]) in AR42J tumor-bearing CB17-SCID mice (100 pmol each). Data of [^177^Lu]Lu-DOTA-rhCCK-18 taken from Günther et al. [20]. These data have been determined in our lab under identical conditions

In order to investigate the effect of a SiFA building block paired with a lower number of negative charges in its direct neighborhood on kidney retention, we selected the most favorable compound with regard to in vitro data (displaying both high CCK-2R affinity and low lipophilicity) comprising either a p-SiFA or SiFAlin moiety. Hence, [^177^Lu]Lu-DOTA-rhCCK-70 (apparent *IC*_50_ = 12.6 ± 2.0 nM, log*D*_7.4_ =  − 1.67 ± 0.08), bearing a p-SiFA moiety, and [^177^Lu]Lu-DOTA-rhCCK-91 (apparent *IC*_50_ = 8.6 ± 0.7 nM, log*D*_7.4_ =  − 1.66 ± 0.08), comprising a SiFAlin moiety, were further evaluated in vivo (Fig. 3, Additional file 1: Table S2 and S3) and compared to [^177^Lu]Lu-DOTA-rhCCK-18 [20].

Activity levels in the AR42J tumor xenograft of 12.0 ± 0.8%ID/g and 7.5 ± 1.0%ID/g were found at 24 h p.i. for [^177^Lu]Lu-DOTA-rhCCK-70 and -91, respectively. Furthermore, activity uptake in the kidneys was observed to be low for both compounds (8.4 ± 0.8 and 6.6 ± 0.5%ID/g) evaluated. In addition, activity levels in liver (3.5 ± 1.7 and 2.0 ± 0.1%ID/g) and spleen (1.9 ± 0.6 and 1.0 ± 0.3%ID/g) were observed to be elevated. Activity accumulation in the CCK-2R-expressing stomach was increased (6.2 ± 0.9 and 4.0 ± 1.2%ID/g). Compared to the reference [^177^Lu]Lu-DOTA-rhCCK-18 (25.4 ± 4.7%ID/g, [20]), activity levels in the tumor were decreased 2- to threefold for [^177^Lu]Lu-DOTA-rhCCK-70 and -91, respectively. However, kidney accumulation and retention of the novel compounds was reduced 16- to 20-fold, respectively (*p* < 0.0001), thus resulting in enhanced tumor-to-kidney ratios (0.19 ± 0.01 vs. 1.44 ± 0.14 and 1.14 ± 0.12, respectively).

**Fig. 4:**
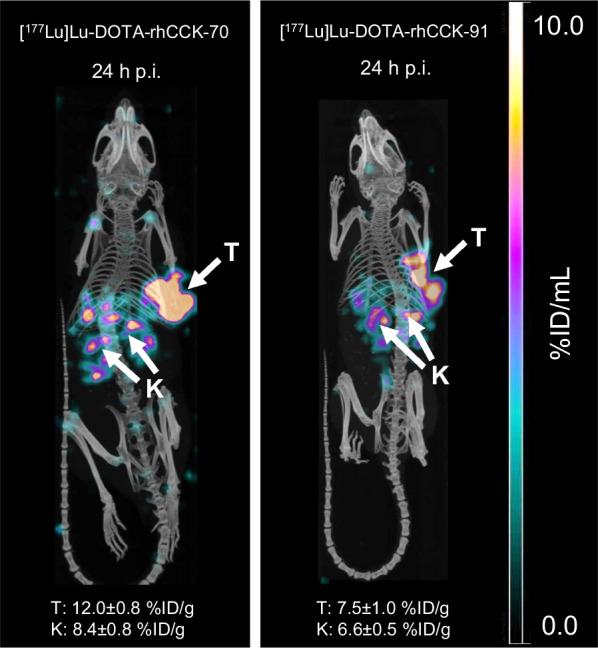
Representative µSPECT/CT images of AR42J tumor-bearing CB17-SCID mice at 24 h p.i. injected either with [^177^Lu]Lu-DOTA-rhCCK-70 (left) or [^177^Lu]Lu-DOTA-rhCCK-91 (right) (100 pmol each). Tumors (T) and kidneys (K) are indicated by white arrows. Mean activity levels in the kidneys (K) and the tumor (T) are shown at the bottom.

µSPECT/CT imaging studies of [^177^Lu]Lu-DOTA-rhCCK-70 and [^177^Lu]Lu-DOTA-rhCCK-91 at 24 h p.i. corroborated the biodistribution data well, revealing high activity levels in the tumor accompanied by reduced activity accumulation in the kidneys (Fig. 4).

## Discussion

In the past few years, the rh concept was successfully implemented for prostate-specific membrane antigen targeted compounds, enabling the generation of chemically identical ligands that are either ^18^F- or ^177^Lu-labeled [19, 28]. These so called “true theranostics” allow for the design of chemical identical pairs, such as ^18^F/^nat^Lu (PET/CT) and ^19^F/^177^Lu (therapy), by combining a chelator as well as a SiFA moiety within the peptide structure. In May 2023, rhPSMA-7.3 (Posluma®) has been approved by the FDA for diagnosis of suspected metastatic as well as recurrent prostate cancer. In addition, clinical trials using rhPSMA-10.1 for therapeutic approaches are ongoing [29–32].

As currently applied CCK-2R-targeted compounds bear no option for ^18^F-labeling, we recently transferred the rh concept to minigastrin analogs via introduction of a D-Dap(p-SiFA) moiety into the peptide structure of DOTA-PP-F11N [8, 20]. The most promising rh-based minigastrin analog, [^18/19^F]F-[^177/nat^Lu]Lu-DOTA-rhCCK-18, displayed decelerated clearance kinetics accompanied by high activity levels in the tumor at 1 and 24 h p.i., rendering this compound a valuable asset for PET/CT imaging of MTC. However, unfavorably elevated renal activity retention of DOTA-rhCCK-18 [20] might be a limiting factor for radioligand therapy when ^177^Lu-labeled, particularly with regard to the kidney as a dose-limiting organ.

In this study, we wanted to reduce activity retention in the kidneys for our rhCCK ligands while maintaining high activity levels in the tumor. Therefore, we first examined the influence of PEG linkers of various length (4 to 11) on in vitro properties of rh-based minigastrin analogs. Moreover, the influence of differently charged SiFA moieties e.g., p-SiFA (neutral), SiFAlin (positively charged) and SiFA-ipa (negatively charged) on overall lipophilicity was evaluated. In addition, lipophilicity of compounds with and without a D-γ-Glu unit in direct proximity to the D-Dap(p-SiFA) unit were evaluated.

Displaying apparent *IC*_50_ values of 12 to 16 nM, no trend on CCK-2R affinity was observed for peptides comprising different PEG linker lengths (4 to 11; [^nat^Lu]Lu-DOTA-rhCCK-67, -68, -70, -73, -74 and -76). Compounds that contain an additional D-γ-Glu moiety in proximity to the SiFA building block ([^nat^Lu]Lu-68, -70 and -76) revealed similar apparent *IC*_50_ values (12 to 16 nM) to their counterparts lacking said entity ([^nat^Lu]Lu-67, -73 and -74). In contrast, substitution of p-SiFA by SiFA-ipa led to significantly elevated apparent *IC*_50_ values (*p* < 0.0001, [^nat^Lu]Lu-DOTA-rhCCK-69: 42.1 ± 1.7 nM and -72: 52.3 ± 6.3 nM), suggesting a low tolerability towards negative charges at the SiFA moiety. Replacing p-SiFA by a positively charged SiFAlin unit had a positive impact on CCK-2R affinity of compounds comprising a (PEG)_4_ chain ([^nat^Lu]Lu-DOTA-rhCCK -90 and -91: apparent *IC*_50_ = 8 to 10 nM), whereas no influence as well as a negative influence on apparent *IC*_50_ values of peptides containing a (PEG)_7_ and (PEG)_11_ chain ([^nat^Lu]Lu-DOTA-rhCCK-71 and -75: apparent *IC*_50_ = 13 to 19 nM), respectively, was observed.

Compared to the reference [^nat^Lu]Lu-DOTA-rhCCK-18 (apparent *IC*_50_ = 4.71 ± 0.62 nM, [20]), all compounds evaluated in this study revealed increased apparent *IC*_50_ values (8 to 53 nM), indicating a negative impact of the reduction of negative charges within the linker sequence on CCK-2R affinity. However, the previously published compound [^177^Lu]Lu-(*R*)-DOTAGA-rhCCK-16 ((*R*)-DOTAGA-D-Dap(p-SiFA)-(D-γ-Glu)_6_-Ala-Tyr-Gly-Trp-Nle-Asp-Phe-NH_2_), displaying an apparent *IC*_50_ value of 20.4 ± 2.7 nM, revealed high activity levels in the tumor (18.0 ± 0.7%ID/g) at 24 h p.i. in AR42J tumor-bearing CB17-SCID mice, which could be attributed to its increased HSA binding and circulation in the blood, thus increasing the window for the delivery of the radiolabeled compound [8]. As all compounds evaluated in this study also comprised a SiFA building block and revealed high HSA binding, we considered apparent *IC*_50_ values < 20 nM sufficient for further in vivo evaluation.

In line with previous findings, the additional negative charge of a D-γ-Glu moiety in proximity to the SiFA building block resulted in a lower lipophilicity for the respective peptides. Moreover, it could be observed that the impact of PEG linker length on overall lipophilicity of CCK2R-targeted compounds was low, which corroborates the data from Novak et al. [33]. Furthermore, peptides comprising a SiFA-ipa moiety displayed the most favorable lipophilicity (log*D*_7.4_: − 2.3 to − 2.1). Surprisingly, the additional positive charge of the SiFAlin moiety had no impact on lipophilicity for [^177^Lu]Lu-DOTA-rhCCK-71, [^177^Lu]Lu-DOTA-rhCCK-90 and [^177^Lu]Lu-DOTA-rhCCK-91**,** or even led to increased log*D*_7.4_ values ([^177^Lu]Lu-DOTA-rhCCK-75). We assume that this is attributed to the change of the overall charge of the peptides. Namely, introduction of a positive charge via a SiFAlin moiety into the mainly negatively charged minigastrin analog would lead to a decreased overall charge and thus, a less beneficial impact on lipophilicity. Except [^177^Lu]Lu-DOTA-rhCCK-69 and -72, each comprising a SiFA-ipa moiety, no other peptides evaluated within this study displayed log*D*_7.4_ values within a range of –3 to –2, which we usually consider ideal due to the favorable pharmacokinetic properties observed for several compounds in the field of nuclear medicine, among those [^177^Lu]Lu-DOTA-rhCCK-18 (log*D*_7.4_ = − 2.69 ± 0.06 [20]) and [^177^Lu]Lu-DOTA-MGS5 (log*D*_7.4_ = − 2.21 ± 0.08 [34]) in case of CCK-2R ligands. However, currently clinically applied radiotracers e.g., [^177^Lu]Lu-Pentixather (log*D*_7.4_ = − 1.8 ± 0.2 [35]) for C-X-C chemokine receptor type 4 targeting and [^177^Lu]Lu-NeoBOMB1 (log*D*_7.4_ = − 0.57 ± 0.03 [27]) addressing the gastrin releasing peptide receptor, also display elevated lipophilicity (log*D*_7.4_ values were evaluated in our group using a comparable experimental setup). Therefore, we decided to extend the range of suitable log*D*_7.4_ values from − 2 to − 1.5, bearing in mind that enhanced hepatic accumulation and thus, effects on the biodistribution profile could occur.

HSA binding was observed to be high (85–95%) for all rhCCK derivatives tested. In comparison, the reference compound [^nat^Lu]Lu-DOTA-rhCCK-18 (87%) displayed a similar HSA interaction. All compounds evaluated comprise a SiFA building block within their peptide structure, which was reported to increase HSA binding [29]. Elevated HSA binding is usually associated with a decelerated activity clearance and prolonged circulation of the compound in the blood stream, which can result in increased activity accumulation in the tumor [36–38]. This corroborates the observed tumor accumulation and retention for previous rhCCK derivatives, such as [^177^Lu]Lu-(*R*)-DOTAGA-rhCCK-16 and [^177^Lu]Lu-DOTA-rhCCK-18. Therefore, we anticipated a similarly beneficial effect on our novel rhCCK ligands.

In order to evaluate the influence of the different SiFA moieties paired with a reduced number of negative charges within the linker sequence of rhCCK derivatives on in vivo performance, particularly with regard to kidney retention, we decided to further investigate both [^177^Lu]Lu-DOTA-rhCCK-70 (apparent *IC*_50_ = 12.6 ± 2.0 nM, log*D*_7.4_ = − 1.67 ± 0.08) and [^177^Lu]Lu-DOTA-rhCCK-91 (apparent *IC*_50_ = 8.7 ± 0.7 nM, log*D*_7.4_ = − 1.66 ± 0.08) at 24 h p.i. in AR42J tumor-bearing mice, since both displayed acceptable CCK-2R affinity and lipophilicity. Compounds comprising a SiFA-ipa building block were excluded from further in vivo studies due to their insufficient CCK-2R affinity.

Biodistribution profiles of [^177^Lu]Lu-DOTA-rhCCK-70 and -91 confirmed our assumption that synergistic effects between the multiple negative charges of the poly-D-γ-glutamate linker section and the D-Dap(p-SiFA) building block led to elevated activity levels in the kidneys observed for earlier generations of rh-based minigastrin analogs, such as [^177^Lu]Lu-(*R*)-DOTAGA-rhCCK-16 and [^177^Lu]Lu-DOTA-rhCCK-18. In line with previously published data on minigastrin analogs [21, 22], the reduction of negative charges within the linker section from eight to two via substitution of D-γ-Glu moieties by PEG_X_ chains led to significantly decreased activity uptake in the kidneys for both [^177^Lu]Lu-DOTA-rhCCK-70 and [^177^Lu]Lu-DOTA-rhCCK-91 compared to [^177^Lu]Lu-DOTA-rhCCK-18 (8.4 ± 0.8%ID/g and 6.6 ± 0.5%ID/g vs. 134 ± 18%ID/g, [20], *p* < 0.0001). Hence, distinctly improved tumor-to-kidney ratios were observed for [^177^Lu]Lu-DOTA-rhCCK-70 (1.45 ± 0.12) and [^177^Lu]Lu-DOTA-rhCCK-91 (1.14 ± 0.12) opposed to [^177^Lu]Lu-DOTA-rhCCK-18 (0.19 ± 0.01, [20]).

However, the increased lipophilicity of [^177^Lu]Lu-DOTA-rhCCK-70 and [^177^Lu]Lu-DOTA-rhCCK-91 led to lower overall tumor-to-background ratios, particularly in the liver (3.48 ± 1.66%ID/g and 1.96 ± 0.08%ID/g vs. 0.22 ± 0.01%ID/g) and spleen (1.92 ± 0.60%ID/g and 1.04 ± 0.26%ID/g vs. 0.34 ± 0.09%ID/g). In addition, increased activity levels in the bone were observed, especially for [^177^Lu]Lu-DOTA-rhCCK-70 (2.4 ± 1.5%ID/g) in comparison to our reference compound [^177^Lu]Lu-DOTA-rhCCK-18 (0.20 ± 0.03%ID/g). This can be partly attributed to the ^177^Lu-batch used for in vivo experiments performed within this study. While usually, radiolabeling with our rhCCK derivatives results in < 1% free lutetium-177 (e.g., [^177^Lu]Lu-DOTA-rhCCK-18), in the study presented herein up to 3% of free lutetium-177 was detected, depending on the ^177^Lu-batch used. Thereby, the amount of free lutetium-177 observed was found to correlate with activity levels in the bone, which was observed to be between 0.5 and 4.3%ID/g, resulting in a high standard deviation (± 1.5%).

Worth mentioning, tumor retention was also reduced noticeably for our novel compounds (12.0 ± 0.8%ID/g and 7.5 ± 1.0%ID/g vs. 25.4 ± 4.7%ID/g, 24 h p.i. [20]). We suggest that this is partly due to their reduced CCK-2R affinity. However, although both compounds revealed a higher CCK-2R affinity compared to [^177^Lu]Lu-(*R*)-DOTAGA-rhCCK-16 (*IC*_50_ = 20.4 ± 2.7 nM; activity levels in the tumor: 18.0 ± 0.7%ID/g [8]), significantly lower activity levels were found in the tumor. Therefore, a more detailed investigation on albumin binding has to be completed, as we expect a higher albumin binding and thus, slower activity clearance from the blood for [^177^Lu]Lu-(*R*)-DOTAGA-rhCCK-16 (containing more negative charges in proximity to the SiFA moiety than both [^177^Lu]Lu-DOTA-rhCCK-70 and -91), which results in a prolonged bioavailability and thus, tumor accumulation of said compound. Similar effects on albumin binding were observed in our group for SiFA-comprising PSMA-targeted compounds [29, 39]. Furthermore, overall charge of the peptide is also an important factor, as [^177^Lu]Lu-DOTA-rhCCK-91 displayed a higher CCK-2R affinity, yet decreased activity levels in the tumor compared to [^177^Lu]Lu-DOTA-rhCCK-70. We thus suggest that the positively charged SiFAlin moiety has a negative effect on tumor accumulation, which has to be further elucidated in future studies. While the lower activity levels in the tumor can be attributed to the lower CCK-2R affinity, it was surprising that activity levels in the stomach, physiologically expressing the CCK-2R, were found to be increased and similar for our novel compounds compared to [^177^Lu]Lu-DOTA-rhCCK-18 (70: 6.2 ± 0.9%ID/g and 91: 4.0 ± 1.2%ID/g vs. 4.3 ± 1.1%ID/g, [20]), respectively. Further experiments have to be carried out in future studies to elucidate the nature of this observation.

In summary, we could achieve our goal to design a rh-based minigastrin analog with substantially reduced kidney retention by modifying the linker section with regard to negatively charged residues. However, these pleasing results were accompanied by a negative impact on overall tumor accumulation compared to our internal benchmarks. Worth mentioning, [^177^Lu]Lu-DOTA-rhCCK-70 still revealed comparable, as well as higher activity levels in the tumor at 24 h p.i. than [^177^Lu]Lu-DOTA-MGS5 (11.0 ± 1.2%ID/g, [16]) and [^177^Lu]Lu-DOTA-PP-F11N (1.9 ± 0.8%ID/g, [8]), respectively; two compounds that are currently evaluated in clinical trials [5, 14]. However, kidney retention was observed to be slightly elevated (8.4 ± 0.8 vs. 1.3 ± 0.4 [16] and 3.1 ± 0.6%ID/g [8]), which resulted in decreased tumor-to-kidney ratios compared to [^177^Lu]Lu-DOTA-MGS5 (1.4 ± 0.1 vs. 9.4 ± 3.3 [16]) but still higher tumor-to-kidney ratios compared to [^177^Lu]Lu-DOTA-PP-F11N (1.4 ± 0.1 vs. 0.6 ± 0.3%ID/g). However, decreased tumor-to-stomach (2.0 ± 0.2 vs. 4.4 ± 0.7 [16] and 5.2 ± 2.2%ID/g) ratios, as well as increased lipophilicity (*–*1.67 ± 0.08 vs. − 4.75 ± 0.07 [16] and − 2.21 ± 0.08 [8]) was observed for [^177^Lu]Lu-DOTA-rhCCK-70 when compared to the reference ligands. Particularly tumor-to-stomach ratios are important, as the stomach (apart from the kidneys) is also considered a dose-limiting organ due to the physiological expression of CCK-2R.

A limitation of this study is the lack of internalization studies in vitro to get more insight into uptake pattern of the receptor. Furthermore, competition studies in vivo should be performed in order to examine receptor specificity of tumor as well as stomach uptake of our novel rhCCK derivatives. Moreover, biodistribution studies at several time points should be carried out to assess pharmacokinetics over time. Lastly, metabolic stability should be investigated in order to draw conclusions on the in vivo behavior of the new compounds. These limitations will be addressed for future compounds but were not our main interest in this study, as we primarily aimed to reduce high kidney retention observed for previously developed rhCCK derivatives (DOTA-rhCCK-18) by circumventing the synergistic effect present for compounds that contain several negative charges in proximity of a SiFA building block. Our study could demonstrate that this strategy can significantly improve kidney retention, and that DOTA-rhCCK-70 is indeed a promising lead compound, yet further optimization is necessary to pave the way for a clinical translation of rh-based minigastrin analogs for theranostic applications.

## Conclusion

In this study we could demonstrate that a reduction of negative charges within the linker section of rh-based minigastrin analogs via substitution of (D-γ-Glu)_8_ by PEG moieties of various length led to a noticeably lower activity uptake in the kidneys compared with previous rh-based CCK-2R-targeted compounds. However, lower tumor accumulation and thus, overall tumor-to-background ratios in all organs apart from the kidneys were also observed, demanding further optimization of the most promising compound from this study with regard to target affinity, lipophilicity and biodistribution profile.

### Supplementary Information

**Additional file 1.** Characterization of all CCK-2R-targeted compounds (**Figure S1-S12**) evaluated in this work, as well as additional information on the synthesis of the SiFA-ipa building block, labeling procedures, in vitro experiments, as well as CCK-2R affinity, lipophilicity, and human serum albumin binding data (**Table S1**), biodistribution data (Table S2) and tumor-to-background ratios (**Table S3**).

## Data Availability

Data is contained within the article and Additional file 1.

## Abbreviations

A_m_: Molar activities
CCK-2R: Cholecystokinin-2 receptor
Dap: 2,3-Diaminopropionic acid
DOTA: 1,4,7,10-Tetraazacyclododecan-1,4,7,10-tetracetic acid
DOTA-CCK-66: DOTA-D-γ-Glu-(PEG)_3_-Trp-(*N*-Me)Nle-Asp-1-Nal-NH_2_
DOTA-MGS5: DOTA-D-Glu-Ala-Tyr-Gly-Trp-(*N*-Me)Nle-Asp-1-Nal-NH_2_
DOTA-PP-F11N: DOTA-(D-Glu)_6_-Ala-Tyr-Gly-Trp-Nle-Asp-Phe-NH_2_
DOTA-rhCCK-18: (DOTA-D-Dap(p-SiFA)-(D-γ-Glu)_8_-Ala-Tyr-Gly-Trp-Nle-Asp-Phe-NH_2_)
ESI–MS: Electro-spray ionization mass spectrometry
Fmoc: 9-Fluorenylmethoxycarbonyl
HSA: Human serum albumin
HPAC: High performance affinity chromatography
MTC: Medullary thyroid carcinoma
PEG: Polyethylene glycol
PET: Positron emission tomography
radio-TLC: Radio-thin layer chromatography
RCP: Radiochemical purity
RCY: Radiochemical yield
rh: Radiohybrid
RP-HPLC: Reversed-phase high performance liquid chromatography
SiFA: Silicon-based fluoride acceptor
SPPS: Solid-phase peptide synthesis
vs: Versus

## References

[1] Ball DW, Wartofsky L, Van Nostrand D (2006). Clinical aspects of medullary thyroid carcinoma. Thyroid cancer: a comprehensive guide to clinical management.

[2] Araque KA, Gubbi S, Klubo-Gwiezdzinska J (2020). Updates on the management of thyroid cancer. Horm Metab Res.

[3] Hundahl SA, Fleming ID, Fremgen AM, Menck HR (1998). A national cancer data base report on 53,856 cases of thyroid carcinoma treated in the US 1985–1995. Cancer.

[4] Reubi JC, Waser B (1996). Unexpected high incidence of cholecystokinin-B/gastrin receptors in human medullary thyroid carcinomas. Int J Cancer.

[5] Rottenburger C, Nicolas GP, McDougall L, Kaul F, Cachovan M, Vija AH (2020). Cholecystokinin 2 receptor agonist (177)Lu-PP-F11N for radionuclide therapy of medullary thyroid carcinoma: results of the lumed phase 0a study. J Nucl Med.

[6] Sauter AW, Mansi R, Hassiepen U, Muller L, Panigada T, Wiehr S (2019). Targeting of the cholecystokinin-2 receptor with the minigastrin analog (177)Lu-DOTA-PP-F11N: does the use of protease inhibitors further improve in vivo distribution?. J Nucl Med.

[7] Lezaic L, Erba PA, Decristoforo C, Zaletel K, Mikolajczak R, Maecke H (2023). [(111)In]In-CP04 as a novel cholecystokinin-2 receptor ligand with theranostic potential in patients with progressive or metastatic medullary thyroid cancer: final results of a GRAN-T-MTC phase I clinical trial. Eur J Nucl Med Mol Imaging.

[8] Holzleitner N, Günther T, Beck R, Lapa C, Wester HJ (2022). Introduction of a SiFA moiety into the D-glutamate chain of DOTA-PP-F11N results in radiohybrid-based CCK-2R-targeted compounds with improved pharmacokinetics in vivo. Pharmaceuticals.

[9] Grob NM, Schibli R, Béhé M, Mindt TL (2021). Improved tumor-targeting with peptidomimetic analogs of minigastrin 177Lu-PP-F11N. Cancers.

[10] Maina T, Konijnenberg MW, KolencPeitl P, Garnuszek P, Nock BA, Kaloudi A (2016). Preclinical pharmacokinetics, biodistribution, radiation dosimetry and toxicity studies required for regulatory approval of a phase I clinical trial with (111)In-CP04 in medullary thyroid carcinoma patients. Eur J Pharm Sci.

[11] Gubbi S, Koch CA, Klubo-Gwiezdzinska J (2022). Peptide receptor radionuclide therapy in thyroid cancer. Front Endocrinol.

[12] Mansi R, Fani M (2021). Radiolabeled peptides for cancer imaging and therapy: from bench-to-bedside. Chimia.

[13] Klingler M, Summer D, Rangger C, Haubner R, Foster J, Sosabowski J (2019). DOTA-MGS5, a new cholecystokinin-2 receptor-targeting peptide analog with an optimized targeting profile for theranostic use. J Nucl Med.

[14] von Guggenberg E, Uprimny C, Klinger M, Warwitz B, Sviridenko A, Bayerschmidt S, Di Santo G, Virgolini IJ (2023). Preliminary clinical experience with cholecystokinin-2 receptor PET/CT using the 68Ga-labeled minigastrin analog DOTA-MGS5 in patients with medullary thyroid cancer. J Nucl Med.

[15] Uprimny C, von Guggenberg E, Svirydenka A, Mikołajczak R, Hubalewska-Dydejczyk A, Virgolini IJ (2021). Comparison of PET/CT imaging with [(18)F]FDOPA and cholecystokinin-2 receptor targeting [(68)Ga]Ga-DOTA-MGS5 in a patient with advanced medullary thyroid carcinoma. Eur J Nucl Med Mol Imaging.

[16] Günther T, Holzleitner N, Viering O, Beck R, Wienand G, Dierks A (2024). Preclinical evaluation of minigastrin analogs and proof-of-concept [(68)Ga]Ga-DOTA-CCK-66 PET/CT in 2 patients with medullary thyroid cancer. J Nucl Med.

[17] Viering O, Günther T, Holzleitner N, Dierks A, Wienand G, Pfob CH, et al. CCK(2) receptor-targeted PET/CT in medullary thyroid cancer using [(68)Ga]Ga-DOTA-CCK-66. J Nucl Med. 2023. 10.2967/jnumed.123.266380. 10.2967/jnumed.123.266380 37918864

[18] Alauddin MM (2012). Positron emission tomography (PET) imaging with (18)F-based radiotracers. Am J Nucl Med Mol Imaging.

[19] Wurzer A, Di Carlo D, Schmidt A, Beck R, Eiber M, Schwaiger M (2020). Radiohybrid ligands: a novel tracer concept exemplified by 18F- or 68Ga-labeled rhPSMA inhibitors. J Nucl Med.

[20] Günther T, Holzleitner N, Di Carlo D, Urtz-Urban N, Lapa C, Wester HJ (2023). Development of the first (18)F-labeled radiohybrid-based minigastrin derivative with high target affinity and tumor accumulation by substitution of the chelating moiety. Pharmaceutics.

[21] Mather SJ, McKenzie AJ, Sosabowski JK, Morris TM, Ellison D, Watson SA (2007). Selection of radiolabeled gastrin analogs for peptide receptor-targeted radionuclide therapy. J Nucl Med.

[22] Kolenc-Peitl P, Mansi R, Tamma M, Gmeiner-Stopar T, Sollner-Dolenc M, Waser B (2011). Highly improved metabolic stability and pharmacokinetics of indium-111-DOTA-gastrin conjugates for targeting of the gastrin receptor. J Med Chem.

[23] Niedermoser S, Chin J, Wängler C, Kostikov A, Bernard-Gauthier V, Vogler N (2015). In vivo evaluation of 18F-SiFAlin–modified TATE: a potential challenge for 68Ga-DOTATATE, the clinical gold standard for somatostatin receptor imaging with PET. J Nucl Med.

[24] Iovkova L, Wängler B, Schirrmacher E, Schirrmacher R, Quandt G, Boening G (2009). Para-Functionalized Aryl-di-tert-butylfluorosilanes as Potential labeling synthons for 18F radiopharmaceuticals. Chem Eur J.

[25] Valko K, Nunhuck S, Bevan C, Abraham MH, Reynolds DP (2003). Fast gradient HPLC method to determine compounds binding to human serum albumin. Relationships with octanol/water and immobilized artificial membrane lipophilicity. J Pharm Sci.

[26] Yamazaki K, Kanaoka M (2004). Computational prediction of the plasma protein-binding percent of diverse pharmaceutical compounds. J Pharm Sci.

[27] Guenther T, Deiser S, Felber V, Beck R, Wester HJ (2022). Substitution of L-tryptophan by a-methyl-L-tryptophan in 177Lu-RM2 results in 177Lu-AMTG, a high-affinity gastrin-releasing peptide receptor ligand with improved in vivo stability. J Nucl Med.

[28] Wurzer A, Parzinger M, Konrad M, Beck R, Gunther T, Felber V (2020). Preclinical comparison of four [(18)F, (nat)Ga]rhPSMA-7 isomers: influence of the stereoconfiguration on pharmacokinetics. EJNMMI Res.

[29] Wurzer A, Kunert J-P, Fischer S, Felber V, Beck R, Rose FD (2022). Synthesis and preclinical evaluation of 177Lu-labeled radiohybrid PSMA ligands for endoradiotherapy of prostate cancer. J Nucl Med.

[30] Rauscher I, Karimzadeh A, Schiller K, Horn T, D'Alessandria C, Franz C (2021). Detection efficacy of (18)F-rhPSMA-7.3 PET/CT and impact on patient management in patients with biochemical recurrence of prostate cancer after radical prostatectomy and prior to potential salvage treatment. J Nucl Med.

[31] Schuster DM (2022). Detection rate of 18F-rhPSMA-7.3 PET in patients with suspected prostate cancer recurrence: results from a phase 3, prospective, multicenter study (SPOTLIGHT). J Clin Oncol.

[32] Bundschuh RA, Pfob CH, Wienand G, Dierks A, Kircher M, Lapa C (2023). 177 Lu-rhPSMA-10.1 Induces tumor response in a patient with mCRPC after PSMA-directed radioligand therapy with 177 Lu-PSMA-I&T. Clin Nucl Med.

[33] Novak D, Tomašič T, Krošelj M, Javornik U, Plavec J, Anderluh M (2021). Radiolabelled CCK(2) R antagonists containing PEG linkers: design. Synth Eval ChemMedChem.

[34] Holzleitner N, Günther T, Daoud-Gadieh A, Lapa C, Wester H-J (2023). Investigation of the structure-activity relationship at the N-terminal part of minigastrin analogs. EJNMMI Res.

[35] Osl T, Schmidt A, Schwaiger M, Schottelius M, Wester HJ (2020). A new class of PentixaFor- and PentixaTher-based theranostic agents with enhanced CXCR4-targeting efficiency. Theranostics.

[36] Umbricht CA, Benešová M, Schibli R, Müller C (2018). Preclinical development of novel PSMA-targeting radioligands: modulation of albumin-binding properties to improve prostate cancer therapy. Mol Pharm.

[37] Kelly JM, Amor-Coarasa A, Ponnala S, Nikolopoulou A, Clarence Williams J, DiMagno SG (2019). Albumin-binding PSMA ligands: implications for expanding the therapeutic window. J Nucl Med.

[38] Deberle LM, Benešová M, Umbricht CA, Borgna F, Büchler M, Zhernosekov K (2020). Development of a new class of PSMA radioligands comprising ibuprofen as an albumin-binding entity. Theranostics.

[39] Kunert JP, Fischer S, Wurzer A, Wester HJ (2022). Albumin-mediated size exclusion chromatography: the apparent molecular weight of PSMA radioligands as novel parameter to estimate their blood clearance kinetics. Pharmaceuticals.

