# Supplementary material for: Significant reduction of activity retention in the kidneys via optimized linker sequences in radiohybrid-based minigastrin analogs
Source: EJNMMI Res. 2024 Mar 2;14:23. doi: 10.1186/s13550-024-01087-5 (PMC10907560; doi:10.1186/s13550-024-01087-5)
Supplement: Supplementary file 1 — Additional file 1. Characterization of all CCK-2R-targeted compounds (Figure S1-S12) evaluated in this work, as well as additional information on the synthesis of the SiFA-ipa building block, labeling procedures, in vitro experiments, as well as CCK-2R affinity, lipophilicity, and human serum albumin binding data (Table S1), biodistribution data (Table S2) and tumor-to-background ratios (Table S3). [file 13550_2024_1087_MOESM1_ESM.docx]

**Significant Reduction of Activity Retention in the Kidneys via Optimized Linker Sequences in Radiohybrid-Based Minigastrin Analogs**

**- Supplementary Materials -**

Nadine Holzleitner^1,^*, Sebastian Fischer^1^, Isabel Maniyankerikalam^1^, Roswitha Beck^1^, Constantin Lapa^2,3^, Hans-Jürgen Wester^1^ and Thomas Günther^1,^*

^1^ TUM School of Natural Sciences, Department of Chemistry, Chair of Pharmaceutical Radiochemistry, Technical University of Munich, Garching, Germany

^2^ Nuclear Medicine, Faculty of Medicine, University of Augsburg, Augsburg, Germany

^3^ Bavarian Cancer Research Center (BZKF), Bavaria, Germany

* correspondence: [thomas.guenther@tum.de](mailto:thomas.guenther@tum.de) (T.G.) and [nadine.holzleitner@tum.de](mailto:nadine.holzleitner@tum.de) (N.H.)

**Corresponding co-authors:**

Thomas Günther and Nadine Holzleitner

Phone: +49.89.289.12203

Technical University of Munich,

Chair of Pharmaceutical Radiochemistry,

Walther-Meissner-Str. 3

85748 Garching

GERMANY

Fax: +49.89.289.12204

E-Mail: [thomas.guenther@tum.de](mailto:thomas.guenther@tum.de) and [nadine.holzleitner@tum.de](mailto:nadine.holzleitner@tum.de)

ORCID-ID: [0000-0002-7412-0297](file:///E:\Nadine%20Holzleitner\CCK\Paper%20CCK\Paper%205%20rhCCK\0000-0002-7412-0297) (TG) and [0000-0001-8258-3526](https://orcid.org/0000-0001-8258-3526) (NH)

**General Information**

Analytical and preparative reversed-phase high performance liquid chromatography (RP-HPLC) were performed using Shimadzu gradient systems (Shimadzu Deutschland GmbH, Neufahrn, Germany), each equipped with a SPD-20A UV/Vis detector (λ = 220 and 254 nm). Different gradients of MeCN (0.1% TFA, 2 or 5% H_2_O for analytical or preparative application, respectively) in H_2_O (0.1% TFA) were used as eluents for all RP-HPLC operations.

For analytical measurements, a MultoKrom 100-5 C18 (150 mm × 4.6 mm) column (CS Chromatographie Service GmbH, Langerwehe, Germany) was used at a flow rate of 1 mL/min. Both, specific gradients and the corresponding retention times *t*_R_ as well as the capacity factor K’ are cited in the text.

Preparative RP-HPLC purification was performed using a MultoKrom 100-5 C18 (250 mm × 20 mm) column (CS Chromatographie GmbH, Langerwehe, Germany) at a constant flow rate of 10 mL/min.

Lyophilization was accomplished using an Alpha 1‑2 LDplus lyophilizer (Martin Christ Gefriertrocknungsanlagen GmbH, Osterode am Harz, Deutschland) combined with a RZ‑2 vacuum pump (Vacuubrand GmbH & Co KG, Olching, Germany).

Analytical and preparative radio RP-HPLC was performed using a MultoKrom 100-5 C18 (5 μm, 125 × 4.6 mm) column (CS Chromatographie GmbH, Langerwehe, Germany). A HERM LB 500 NaI scintillation detector (Berthold Technologies, Bad Wildbad, Germany) was connected to the outlet of the UV photometer for the detection of radioactivity.

Radioactive samples were measured by a WIZARD^2®^ 2480 Automatic *γ*-counter (Perkin Elmer Inc., Waltham, MA, USA).

**Analytical Data of ^nat/177^Lu-labeled Minigastrin Analogs**

**
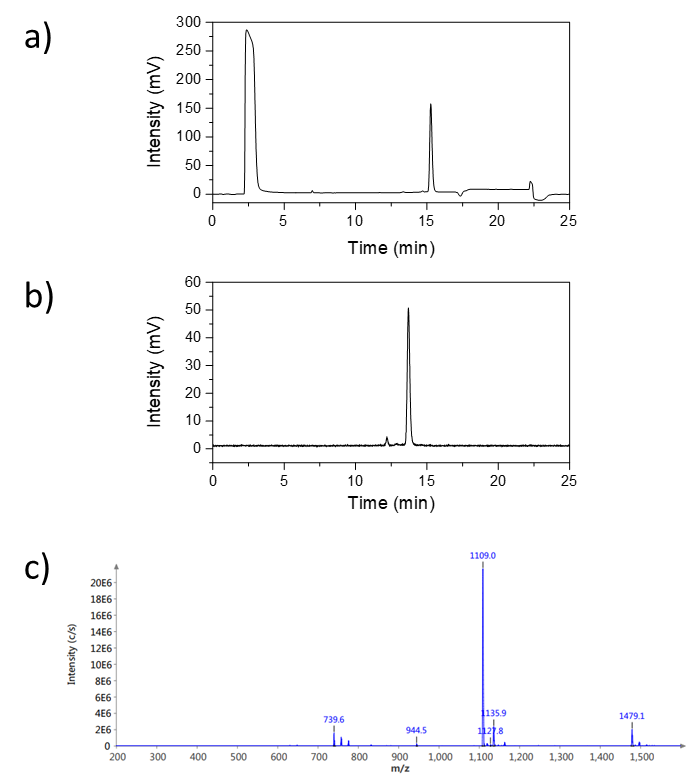
**

**Figure S1.** Confirmation of peptide identity and integrity for **a**) [^nat^Lu]Lu-DOTA-rhCCK-67 and **b**) [^177^Lu]Lu-DOTA-rhCCK-67 as analyzed by analytical (radio-)RP-HPLC 10→70% MeCN in H_2_O + 0.1% TFA in 15 min). **c**) Mass spectrum of [^nat^Lu]Lu-DOTA-rhCCK-67.

***[^nat^Lu]Lu-DOTA-rhCCK-67***: RP-HPLC (10→70% MeCN in H_2_O with 0.1% TFA, 15 min, λ = 220 nm): *t*_R_ = 15.3 min, K’ = 8.07; MS (ESI, positive): m/z calculated for C_98_H_145_FLuN_15_O_29_: 2218.0, found: m/z = 1109.0 [M+2H]^2+^.

**
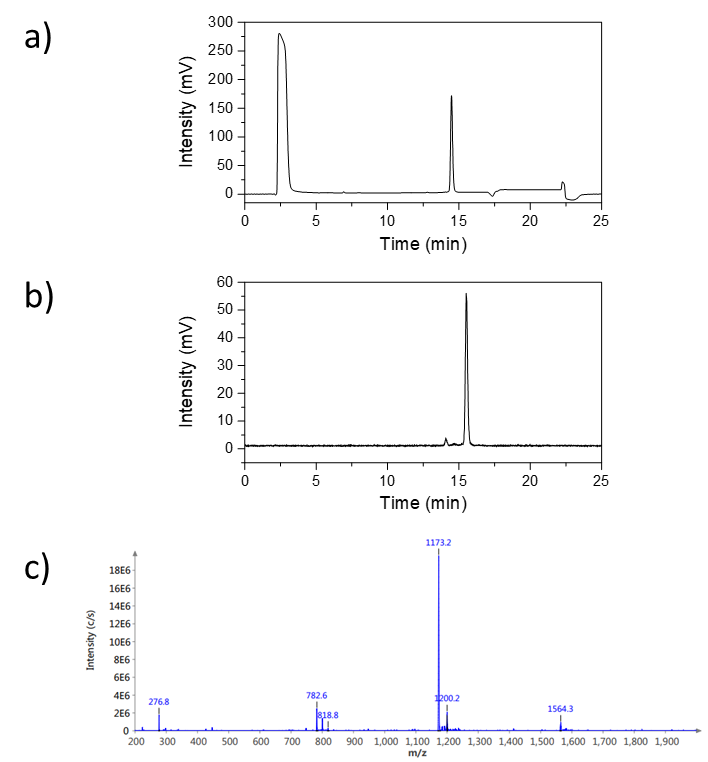
**

**Figure S2.** Confirmation of peptide identity and integrity for **a**) [^nat^Lu]Lu-DOTA-rhCCK-68 and **b**) [^177^Lu]Lu-DOTA-rhCCK-68 as analyzed by analytical (radio-)RP-HPLC (10→70% MeCN in H_2_O + 0.1% TFA in 15 min). **c**) Mass spectrum of [^nat^Lu]Lu-DOTA-rhCCK-68.

***[^nat^Lu]Lu-DOTA-rhCCK-68***: RP-HPLC (10→70% MeCN in H_2_O with 0.1% TFA, 15 min, λ = 220 nm) *t*_R_ = 14.5 min, K’ = 7.60; MS (ESI, positive): m/z calculated for C_103_H_152_FLuN_16_O_32_Si: 2348.5, found: m/z = 1173.2 [M+2H]^2+^.

**
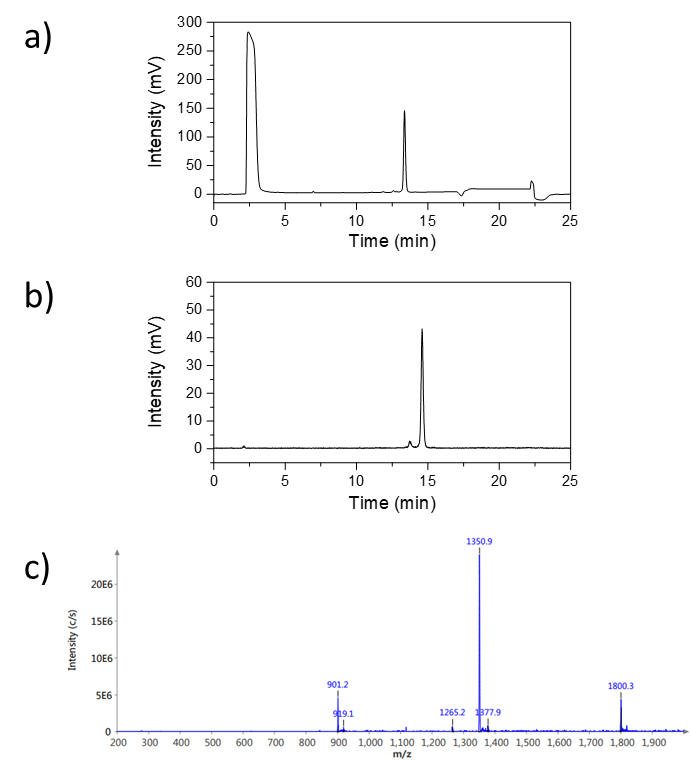
**

**Figure S3.** Confirmation of peptide identity and integrity for **a**) [^nat^Lu]Lu-DOTA-rhCCK-69 and **b**) [^177^Lu]Lu-DOTA-rhCCK-69 as analyzed by analytical (radio-)RP-HPLC (10→70% MeCN in H_2_O + 0.1% TFA in 15 min). **c**) Mass spectrum of [^nat^Lu]Lu-DOTA-rhCCK-69.

***[^nat^Lu]Lu-DOTA-rhCCK-69***: RP-HPLC (10→70% MeCN in H_2_O with 0.1% TFA, 15 min, λ = 220 nm) *t*_R_ = 13.4 min, K’ = 6.94; MS (ESI, positive): m/z calculated for C_118_H_180_FLuN_16_O_41_Si: 2699.17, found: m/z = 1350.9 [M+2H]^2+^, 901.2 [M+3H]^3+^.

**
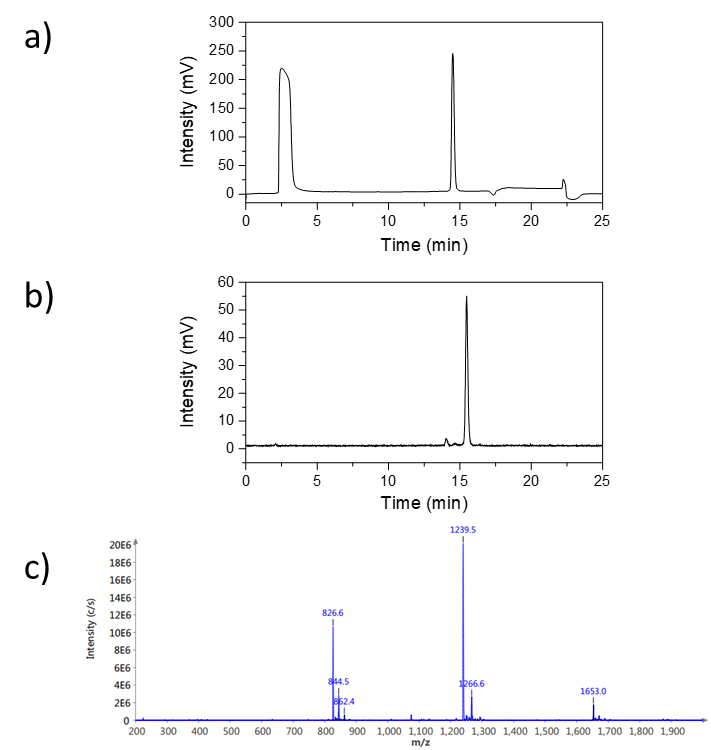
**

**Figure S4.** Confirmation of peptide identity and integrity for **a**) [^nat^Lu]Lu-DOTA-rhCCK-70 and **b**) [^177^Lu]Lu-DOTA-rhCCK-70 as analyzed by analytical (radio-)RP-HPLC (10→70% MeCN in H_2_O + 0.1% TFA in 15 min). **c**) Mass spectrum of [^nat^Lu]Lu-DOTA-rhCCK-70.

***[^nat^Lu]Lu-DOTA-rhCCK-70***: RP-HPLC (10→70% MeCN in H_2_O with 0.1% TFA, 15 min, λ = 220 nm) *t*_R_ = 14.8 min, K’ = 6.40; MS (ESI, positive): m/z calculated for C_109_H_164_FLuN_16_O_35_Si: 2479.1, found: m/z = 1239.5 [M+2H]^2+^, 826.6 [M+3H]^3+^.


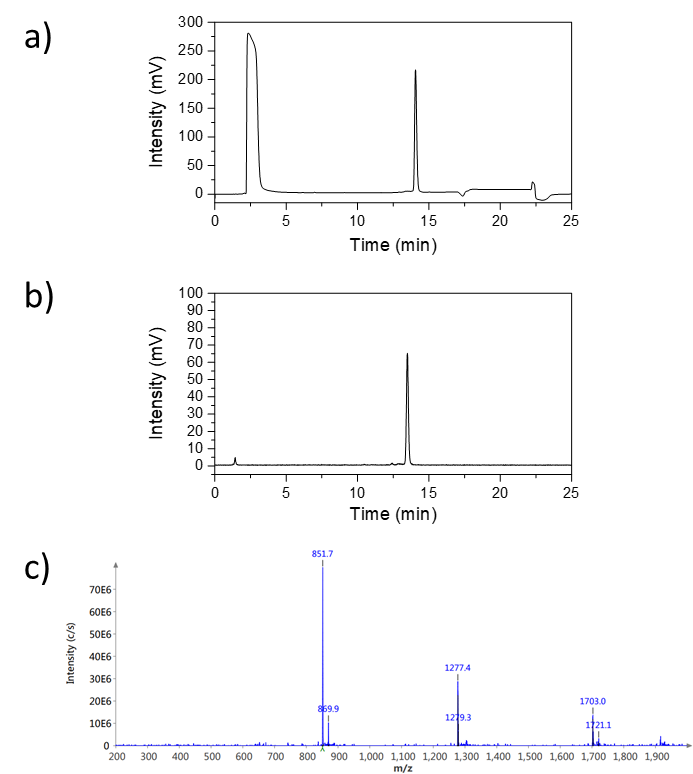


**Figure S5.** Confirmation of peptide identity and integrity for **a**) [^nat^Lu]Lu-DOTA-rhCCK-71 and **b**) [^177^Lu]Lu-DOTA-rhCCK-71 as analyzed by analytical (radio-)RP-HPLC (10→70% MeCN in H_2_O + 0.1% TFA in 15 min). **c**) Mass spectrum of [^nat^Lu]Lu-DOTA-rhCCK-71.

***[^nat^Lu]Lu-DOTA-rhCCK-71***: RP-HPLC (10→70% MeCN in H_2_O with 0.1% TFA, 15 min, λ = 220 nm) *t*_R_ = 14.1 min, K’ = 7.36; MS (ESI, positive): m/z calculated for C_113_H_174_FLuN_17_O_35_Si^+^: 2551.2, found: m/z = 1277.4 [M+2H]^2+^, 851.7 [M+3H]^3+^.

**
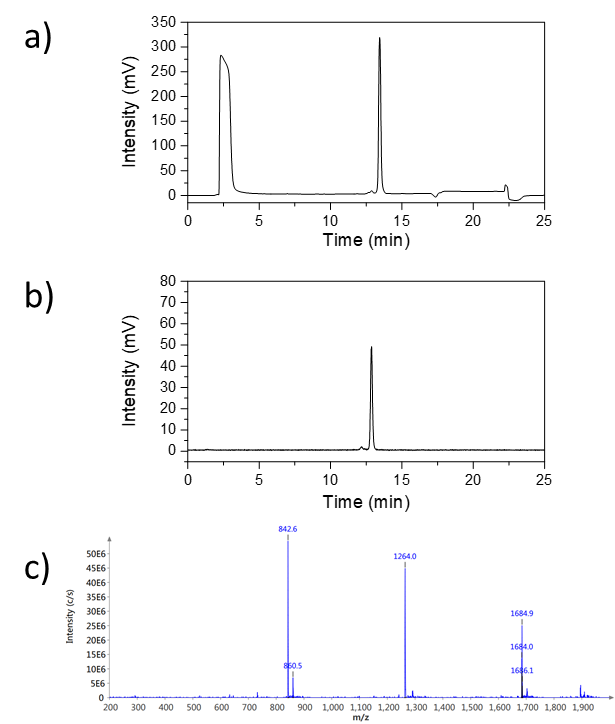
**

**Figure S6.** Confirmation of peptide identity and integrity for **a**) [^nat^Lu]Lu-DOTA-rhCCK-72 and **b**) [^177^Lu]Lu-DOTA-rhCCK-72 as analyzed by analytical (radio-)RP-HPLC (10→70% MeCN in H_2_O + 0.1% TFA in 15 min). **c**) Mass spectrum of [^nat^Lu]Lu-DOTA-rhCCK-72.

***[^nat^Lu]Lu-DOTA-rhCCK-72***: RP-HPLC (10→70% MeCN in H_2_O with 0.1% TFA, 15 min, λ = 220 nm) *t*_R_ = 13.4 min, K’ = 6.94; MS (ESI, positive): m/z calculated for C_110_H_164_FLuN_16_O_37_Si: 2523.1, found: m/z = 1264.0 [M+2H]^2+^, 842.6 [M+3H]^3+^.

**
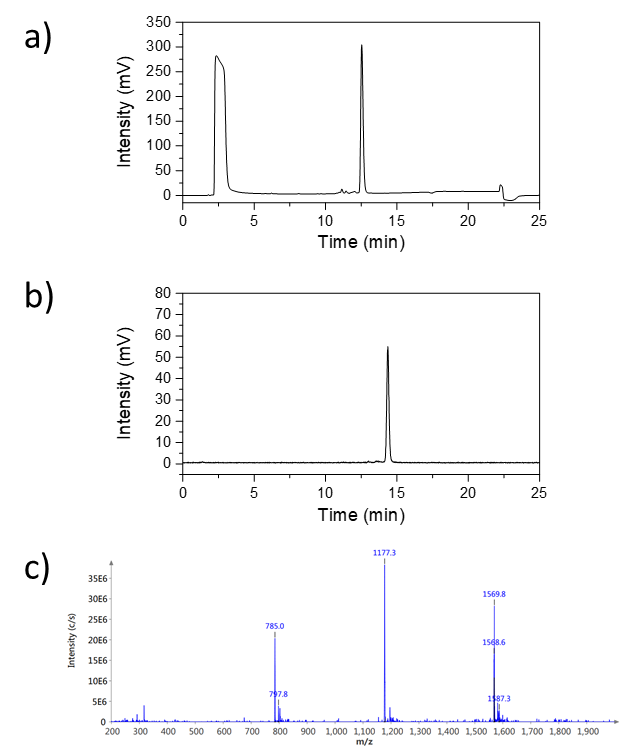
**

**Figure S7.** Confirmation of peptide identity and integrity for **a**) [^nat^Lu]Lu-DOTA-rhCCK-73 and **b**) [^177^Lu]Lu-DOTA-rhCCK-73 as analyzed by analytical (radio-)RP-HPLC (10→90% MeCN in H_2_O + 0.1% TFA in 15 min). **c**) Mass spectrum of [^nat^Lu]Lu-DOTA-rhCCK-73.

***[^nat^Lu]Lu-DOTA-rhCCK-73***: RP-HPLC (10→90% MeCN in H_2_O with 0.1% TFA, 15 min, λ = 220 nm) *t*_R_ = 12.5 min, K’ = 6.41; MS (ESI, positive): m/z calculated for C_104_H_157_FLuN_15_O_32_Si: 2350.0, found: m/z = 1177.3 [M+2H]^2+^, 785.0 [M+3H]^3+^.


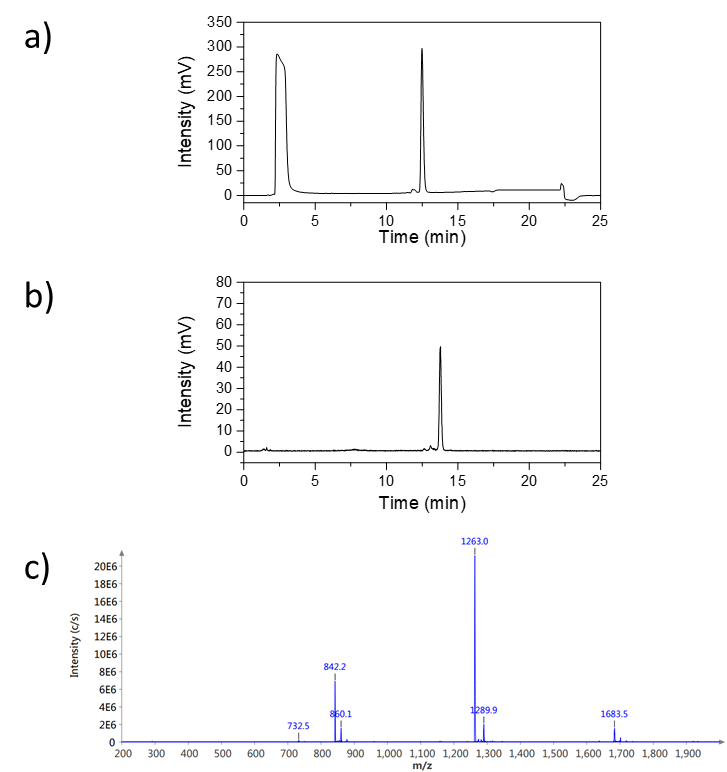


**Figure S8.** Confirmation of peptide identity and integrity for **a**) [^nat^Lu]Lu-DOTA-rhCCK-74 and **b**) [^177^Lu]Lu-DOTA-rhCCK-74 as analyzed by analytical (radio-)RP-HPLC (10→90% MeCN in H_2_O + 0.1% TFA in 15 min). **c**) Mass spectrum of [^nat^Lu]Lu-DOTA-rhCCK-74.

***[^nat^Lu]Lu-DOTA-rhCCK-74***: RP-HPLC (10→90% MeCN in H_2_O with 0.1% TFA, 15 min, λ = 220 nm) *t*_R_ = 12.5 min, K’ = 6.41; MS (ESI, positive): m/z calculated for C_112_H_173_FLuN_15_O_36_Si: 2526.1, found: m/z = 1263.0 [M+2H]^2+^, 842.2 [M+3H]^3+^.

**
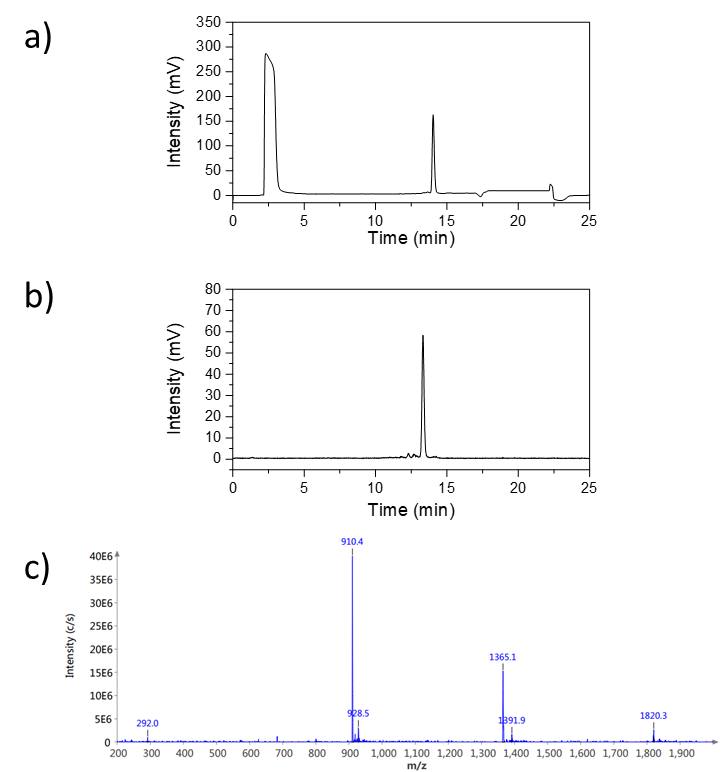
**

**Figure S9.** Confirmation of peptide identity and integrity for **a**) [^nat^Lu]Lu-DOTA-rhCCK-75 and **b**) [^177^Lu]Lu-DOTA-rhCCK-75 as analyzed by analytical (radio-)RP-HPLC (10→70% MeCN in H_2_O + 0.1% TFA in 15 min). **c**) Mass spectrum of [^nat^Lu]Lu-DOTA-rhCCK-75.

***[^nat^Lu]Lu-DOTA-rhCCK-75***: RP-HPLC (10→70% MeCN in H_2_O with 0.1% TFA, 15 min, λ = 220 nm) *t*_R_ = 14.0 min, K’ = 7.30; MS (ESI, positive): m/z calculated for C_121_H_190_FLuN_17_O_39_Si^+^: 2727.3, found: m/z = 1365.1 [M+2H]^2+^, 910.4 [M+3H]^3+^.

**
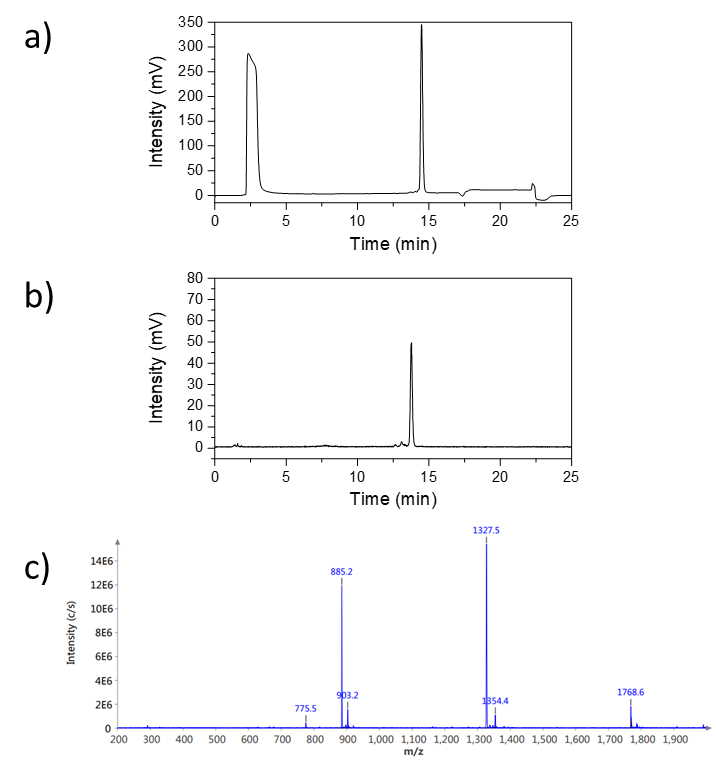
**

**Figure S10.** Confirmation of peptide identity and integrity for **a**) [^nat^Lu]Lu-DOTA-rhCCK-76 and **b**) [^177^Lu]Lu-DOTA-rhCCK-76 as analyzed by analytical (radio-)RP-HPLC (10→70% MeCN in H_2_O + 0.1% TFA in 15 min). **c**) Mass spectrum of [^nat^Lu]Lu-DOTA-rhCCK-76.

***[^nat^Lu]Lu-DOTA-rhCCK-76***: RP-HPLC (10→70% MeCN in H_2_O with 0.1% TFA, 15 min, λ = 220 nm) *t*_R_ = 14.5 min, K’ = 7.60; MS (ESI, positive): m/z calculated for C_118_H_183_FLuN_16_O_39_Si: 2655.2, found: m/z = 1327.5 [M+2H]^2+^, 885.2 [M+3H]^3+^.


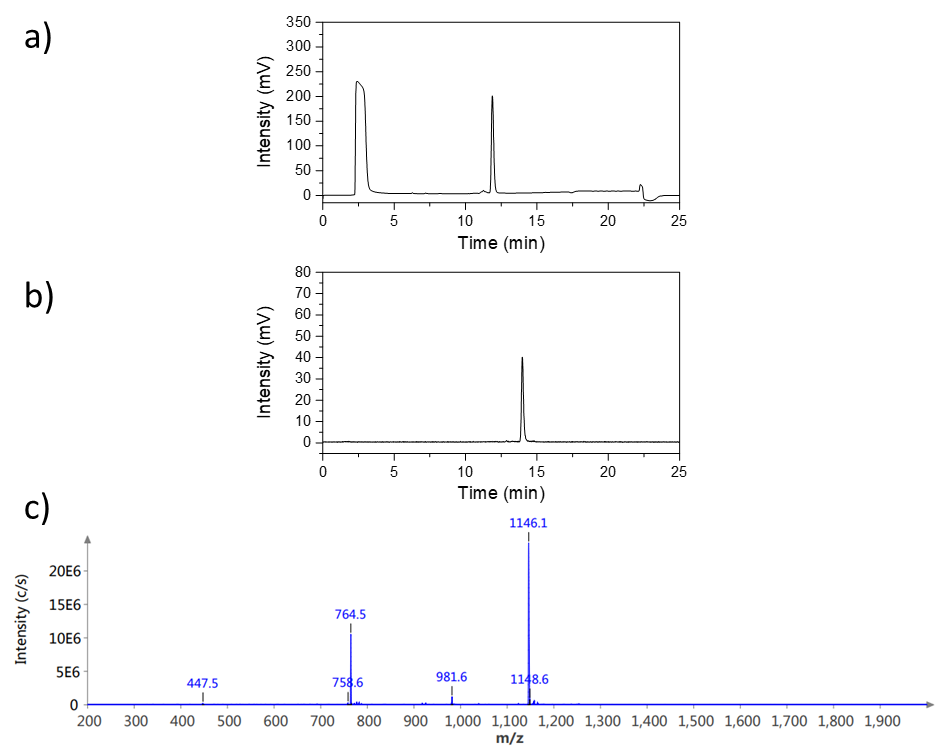


**Figure S11.** Confirmation of peptide identity and integrity for **a**) [^nat^Lu]Lu-DOTA-rhCCK-90 and **b**) [^177^Lu]Lu-DOTA-rhCCK-90 as analyzed by analytical (radio-)RP-HPLC (10→90% MeCN in H_2_O + 0.1% TFA in 15 min). **c**) Mass spectrum of [^nat^Lu]Lu-DOTA-rhCCK-90.

***[^nat^Lu]Lu-DOTA-rhCCK-90***: RP-HPLC (10→90% MeCN in H_2_O with 0.1% TFA, 15 min, λ = 220 nm) *t*_R_ = 11.9 min, K’ = 6.05; MS (ESI, positive): m/z calculated for C_102_H_155_FLuN_16_O_29_Si^+^: 2290.0, found: m/z = 1146.1 [M+2H]^2+^, 764.5 [M+3H]^3+^.

**
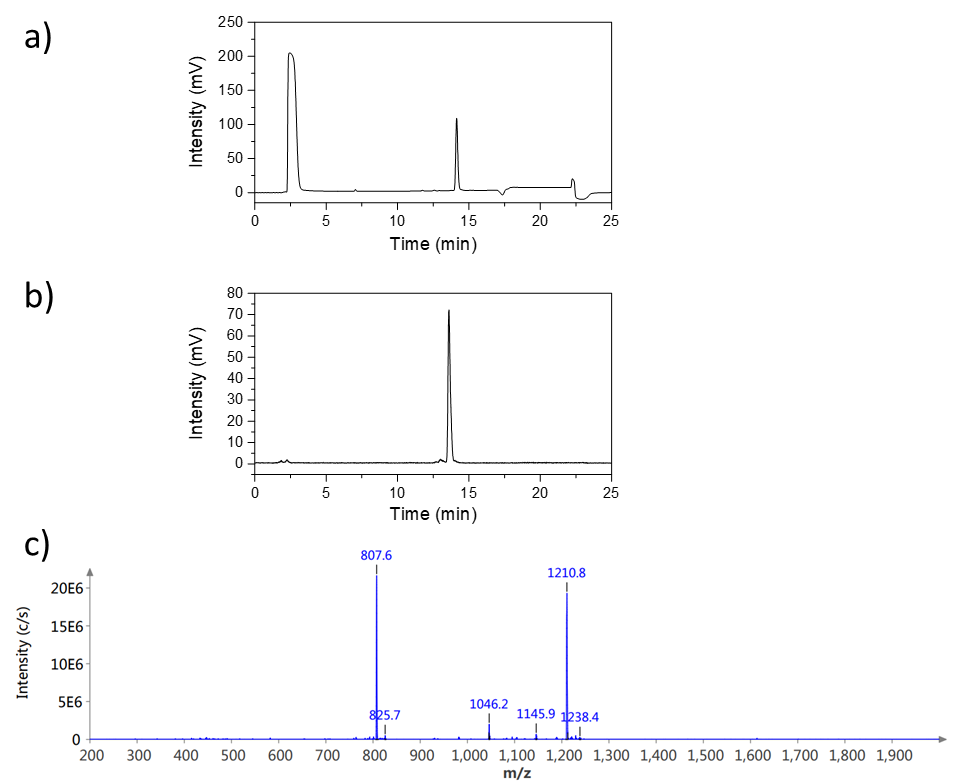
**

**Figure S12.** Confirmation of peptide identity and integrity for **a**) [^nat^Lu]Lu-DOTA-rhCCK-91 and **b**) [^177^Lu]Lu-DOTA-rhCCK-91 as analyzed by analytical (radio-)RP-HPLC (10→70% MeCN in H_2_O + 0.1% TFA in 15 min). **c**) Mass spectrum of [^nat^Lu]Lu-DOTA-rhCCK-91.

***[^nat^Lu]Lu-DOTA-rhCCK-91***: RP-HPLC (10→70% MeCN in H_2_O with 0.1% TFA, 15 min, λ = 220 nm) *t*_R_ = 14.1 min, K’ = 7.36; MS (ESI, positive): m/z calculated for C_107_H_162_FLuN_17_O_32_Si^+^: 2419.1, found: m/z = 1210.8 [M+2H]^2+^, 807.6 [M+3H]^3+^.

**Synthesis of the SiFA-ipa Building Block**

Di-*tert*-butyl(3,5-dimethylphenyl)fluorosilane (i)

1-Bromo-3,5-dimethylbenzene (4.5 g, 25.1 mmol, 1.0 eq.) was dissolved in 73 mL dry THF and cooled to –78°C. Afterwards, *^t^*BuLi (34.7 mL, 55.5 mmol, 1.6 M in pentane, 2.2 eq.) was added dropwise and stirred for 30 min at –78°C. A second solution was prepared by dissolving di-*tert*-butyldifluorosilane (5.0 g, 27.7 mmol, 1.1 eq.) in 49 mL dry THF and cooling it to –78°C. Then, the first solution was added and the reaction mixture was stirred overnight, while slowly warming to room temperature. Addition of 100 mL brine terminated the reaction, aqueous layer was extracted with Et_2_O (3×100 mL), combined organic phases were dried using MgSO_4_ and the solvent was removed under reduced pressure. Di-*tert*-butyl(3,5-dimethylphenyl)fluorosilane (i, 6.6 g, 24.8 mmol, 99 %) was obtained as a colorless solid.

^1^H NMR (500 MHz, CDCl_3_): d (ppm) = 7.19 (s, 2H; H*_o_*), 7.04 (s, 1H; H*_p_*), 2.33 (s, 6 H; CH_3_), 1.06 (s, 18 H; CCH_3_). RP‑HPLC (50 to 100% B in 15 min, 100% B for 10 min): *t_R_* = 16.4 min. K’ = 8.21.

5-(Di-*tert*-butylfluorosilyl)isophthalic acid (SiFA-ipa, ii)

1.1 g Di-*tert*-butyl(3,5-diemethylphenyl)fluorosilane (i, 1.1 g, 4.0 mmol, 1.0 eq.) were dissolved in 17 mL *^t^*BuOH/CH_2_Cl_2_ (*v/v* = 3.5/1) and NaH_2_PO_4_×H_2_O (16 mL, 40 mmol, 2.5 M in H_2_O, 10 eq.) as well as KMnO_4_ (7.6 g, 48 mmol, 12 eq.) were added. Afterwards, the reaction mixture was slowly heated to 75°C and stirred for 24 h. Subsequently, the reaction was terminated by addition of 50 mL saturated aqueous NaSO_3_ solution and residual MnO_2_ was dissolved with 10 mL concentrated HCl. The aqueous phase was extracted with Et_2_O (3×100 mL), the combined organic phases were dried over MgSO_4_ and the solvent was removed under reduced pressure. SiFA-ipa (ii, 1.3 g, 4.0 mmol, >99%) was obtained as a colorless solid.

^1^H NMR (400 MHz, DMSO-D_6_): d (ppm) = 8.53 (t, 1 H, ^4^*J*(^1^H,^1^H) = 1.7 Hz; H_Ar-2_), 8.32 (d, 2 H, ^4^*J*(^1^H,^1^H) = 1.6 Hz; H_Ar-4,-6_), 1.03 (s, 18 H; CH_3_).

^13^C{^1^H} NMR (101 MHz, DMSO-D_6_): d [ppm] = 166.5 (s; COOH), 137.9 (d, ^3^*J*(^13^C,^19^F) = 4 Hz; C_Ar-4,-6_), 134.0 (d, ^2^*J*(^13^C,^19^F) = 14 Hz; C_Ar-5_), 131.4 (s; C_Ar-2_), 130.8 (s; C_Ar‑1,‑3_), 26.8 (s; C*C*H_3_), 19.7 (d, ^2^*J*(^13^C,^19^F) = 12 Hz; *C*CH_3_).

^19^F{^29^Si} NMR (376 MHz, DMSO-D_6_): d (ppm) = –187.1.

^29^Si{^1^H}INEPT NMR (79 MHz, DMSO-D_6_): d (ppm) = 13.8 (d, ^1^*J*(^19^F,^29^Si) = 299 Hz).

RP-HPLC (50→100% MeCN in H_2_O with 0.1% TFA, 15 min, λ = 220 nm) *t*_R_ = 5.7 min, K’ = 2.20; MS (ESI, positive): m/z calculated for C_16_H_23_FO_4_Si: 326.1, found: m/z = 327.2 [M+H]^+^, 309.2 [M–H_2_O+H]^+^.

**Scheme S1:** Synthesis of SiFA-ipa (ii): a) ^t^Bu_2_SiF_2_, ^t^BuLi, −78°C to rt, overnight (THF) b) KMnO_4_, 75°C, 24 h (CH_2_Cl_2_/^t^BuOH/NaH_2_PO_4_×H_2_O buffer).

**Labeling Procedures**

^nat^Lu-Labeling

Quantitative ^nat^Lu-labeling was conducted by addition of a 2.5-fold excess of [^nat^Lu]LuCl_3_ (20 mM in H_2_O) to the peptide precursor (1 mM in DMSO), followed by heating the reaction mixture to 90°C for 15 min. Peptide identity and integrity were confirmed using RP-HPLC and ESI-MS.

^177^Lu-Labeling

^177^Lu-Labeling of the peptide precursor (1 nmol) was performed at 80°C for 10 min in a NaOAc-buffered (1 M, pH = 5.5) hydrochloric acid (0.04 M) solution using [^177^Lu]LuCl_3_ dissolved in hydrochloric acid (0.04 M, 40 GBq/mL) acquired from ITM Isotope Technologies Munich SE (Garching, Germany). After the reaction was finished, sodium ascorbate (1 M in H_2_O, 10 vol-%) was added as radiolysis quencher. Radiochemical purity was determined via radio-RP-HPLC and radio-TLC (instant thin layer chromatography paper impregnated with silica gel (iTLC-SG, Agilent Technologies Inc., Folsom, United States); sodium citrate*1.5 H_2_O (0.1 M)).

***In Vitro* Experiments**

Cell Culture

CCK-2R positive rat pancreatic cancer cells AR42J (CLS GmbH*,* Eppelheim, Germany) were cultivated in monolayers in CELLSTAR^®^ cell culture flask (Greiner Bio-One GmbH, Frickenhausen, Germany) at 37°C in a humidified atmosphere (5% CO_2_). Therefor, a HERAcell 150i-Incubator (Thermo Fisher Scientific Inc., Waltham, United States) was used. RPMI 1640 medium, supplemented with 5 mM L-Gln 5 mL non-essential amino acids (100×) and 10% FCS, was used for cell nutrition. Detachment of the cells for passaging was conducted with a Dulbecco’s PBS solution supplemented with 0.1% EDTA (*v/v*). Cell numbers were determined with a Neubauer hemocytometer (Paul Marienfeld, Lauda-Königshofen, Germany). All operations requiring sterile conditions, were accomplished using a MSC-Advantage safety workbench (Thermo Fisher Scientific Inc., Waltham, United States).

Human Serum Albumin Binding

Human serum albumin binding was determined via high performance affinity chromatography. Therefore, a Chiralpak HSA column (50 × 3 mm, 5 µm, H13H-2433, Daicel, Tokyo, Japan) was used at a constant flow rate of 0.5 ml/min at room temperature. Mobile phase A consisted of a freshly prepared aqueous solution of NH_4_OAc (pH = 6.9, 50 mM) and mobile phase B of isopropanol. Calibration of the column was performed daily prior to experiments, by determining the retention times of nine reference substances displaying a HSA binding from 13 to 99%. HPLC gradient for all compounds tested was 100% A (0 to 3 min), followed by 80% A (3 to 40 min). All substances tested, were dissolved in a mixture of A/B (0.5 mg/mL, 1:1, *v*/*v*). OriginPro 2016G software (Northampton, United States) was used for non-linear regression and data evaluation.

**Table S1.** Affinity, lipophilicity and human serum albumin binding data of the compounds evaluated. Affinity data were determined on AR42J cells (2.0 × 10^5^ cells/well) and [^177^Lu]Lu-DOTA-PP-F11N (0.3 pmol/well) as radiolabeled reference (3 h, 37 °C, RPMI 1640, 5 mM L-Gln, 5 mL non-essential amino acids (100×), 10% FCS + 5% BSA (*v*/*v*)).

| **Peptide** | **apparent *IC*_50_**  (nM) | **log*D*_7.4_** | **HSA**  (%) |
| --- | --- | --- | --- |
| [^nat/177^Lu]Lu-DOTA-rhCCK-67 | 13.0 ± 1.1 | −1.05 ± 0.09 | 92.9 |
| [^nat/177^Lu]Lu-DOTA-rhCCK-68 | 15.4 ± 1.1 | −1.65 ± 0.09 | 91.2 |
| [^nat/177^Lu]Lu-DOTA-rhCCK-69 | 43.1 ± 1.7 | −2.11 ± 0.09 | 84.7 |
| [^nat/177^Lu]Lu-DOTA-rhCCK-70 | 12.6 ± 2.0 | −1.67 ± 0.08 | 89.3 |
| [^nat/177^Lu]Lu-DOTA-rhCCK-71 | 13.3 ± 1.3 | −1.63 ± 0.06 | 89.1 |
| [^nat/177^Lu]Lu-DOTA-rhCCK-72 | 52.3 ± 6.2 | −2.32 ± 0.03 | 89.3 |
| [^nat/177^Lu]Lu-DOTA-rhCCK-73 | 15.6 ± 0.7 | −1.02 ± 0.06 | 90.4 |
| [^nat/177^Lu]Lu-DOTA-rhCCK-74 | 12.1 ± 1.0 | −1.19 ± 0.07 | 87.2 |
| [^nat/177^Lu]Lu-DOTA-rhCCK-75 | 19.2 ± 1.6 | −1.15 ± 0.05 | 90.9 |
| [^nat/177^Lu]Lu-DOTA-rhCCK-76 | 14.2 ± 0.9 | −1.82 ± 0.09 | 86.2 |
| [^nat/177^Lu]Lu-DOTA-rhCCK-90 | 9.9 ± 1.0 | −0.76 ± 0.09 | 94.5 |
| [^nat/177^Lu]Lu-DOTA-rhCCK-91 | 8.6 ± 0.7 | −1.66 ± 0.08 | 90.0 |

**Table S2.** Biodistribution data of [^177^Lu]Lu-DOTA-rhCCK-70 and [^177^Lu]Lu-DOTA-rhCCK-91 in selected organs at 24 h p.i. in AR42J tumor-bearing CB17-SCID mice (100 pmol each). Data are expressed as %ID/g, mean ± SD.

| **organ** | **[^177^Lu]Lu-DOTA-rhCCK-70**  *(n*=4*)* | **[^177^Lu]Lu-DOTA-rhCCK-91**  *(n*=4*)* |
| --- | --- | --- |
| Blood | 0.10 ± 0.04 | 0.04 ± 0.01 |
| Heart | 0.23 ± 0.05 | 0.31 ± 0.07 |
| Lung | 0.39 ± 0.12 | 0.26 ± 0.05 |
| Liver | 3.48 ± 1.66 | 1.96 ± 0.08 |
| Spleen | 1.92 ± 0.60 | 1.04 ± 0.26 |
| Pancreas | 0.24 ± 0.08 | 0.90 ± 0.32 |
| Stomach | 6.21 ± 0.85 | 3.99 ± 1.16 |
| Intestine | 0.48 ± 0.13 | 0.68 ± 0.16 |
| Kidney | 8.37 ± 0.78 | 6.58 ± 0.53 |
| Adrenal | 0.55 ± 0.46 | 0.44 ± 0.09 |
| Muscle | 0.06 ± 0.03 | 0.06 ± 0.01 |
| Bone | 2.40 ± 1.51 | 0.64 ± 0.12 |
| Tumor | 12.0 ± 0.8 | 7.47 ± 1.01 |

**Table S3.** Tumor-to-background ratios of [^177^Lu]Lu-DOTA-rhCCK-70 and [^177^Lu]Lu-DOTA-rhCCK-91 in selected organs at 24 h p.i. in AR42J tumor-bearing CB17-SCID mice (100 pmol each). Data are expressed as %ID/g, mean ± SD.

|  | **[^177^Lu]Lu-DOTA-rhCCK-70**  *(n*=4*)* | **[^177^Lu]Lu-DOTA-rhCCK-91**  *(n*=4*)* |
| --- | --- | --- |
| Blood | 143 ± 49 | 185 ± 32 |
| Heart | 55.4 ± 13.4 | 25.3 ± 5.2 |
| Lung | 33.4 ± 8.53 | 29.3 ± 4.2 |
| Liver | 4.26 ± 1.70 | 3.80 ± 0.37 |
| Spleen | 6.95 ± 2.17 | 7.35 ± 0.75 |
| Pancreas | 55.2 ± 19.6 | 8.98 ± 2.10 |
| Stomach | 1.97 ± 0.24 | 1.95 ± 0.27 |
| Intestine | 27.4 ± 7.7 | 12.3 ± 5.1 |
| Kidney | 1.44 ± 0.12 | 1.14 ± 0.12 |
| Adrenal | 20.5 ± 8.5 | 17.3 ± 1.9 |
| Muscle | 276 ± 189 | 147 ± 65 |
| Bone | 9.48 ± 8.05 | 11.8 ± 0.72 |
